# A systematic review of cognitive interventions for adult patients with brain tumours

**DOI:** 10.1002/cam4.5760

**Published:** 2023-03-07

**Authors:** Matthew A. Kirkman, Justyna O. Ekert, Benjamin H. M. Hunn, Michael S. C. Thomas, Andrew K. Tolmie

**Affiliations:** ^1^ Department of Psychology and Human Development, UCL Institute of Education University College London London UK; ^2^ Department of Neurosurgery, Queen's Medical Centre Nottingham University Hospitals NHS Trust Nottingham UK; ^3^ Wellcome Centre for Human Neuroimaging UCL Queen Square Institute of Neurology London UK; ^4^ Department of Neurosurgery Royal Melbourne Hospital Melbourne Australia; ^5^ Department of Neurosurgery Royal Hobart Hospital Hobart Australia; ^6^ School of Medicine University of Tasmania Hobart Australia; ^7^ Department of Psychological Sciences, Birkbeck University of London London UK

**Keywords:** brain tumour, cognitive outcomes, interventions, pharmacological, rehabilitation

## Abstract

**Background:**

Neurocognitive impairments are common in patients with current or previously treated brain tumours, and such impairments can negatively affect patient outcomes including quality of life and survival. This systematic review aimed to identify and describe interventions used to ameliorate (improve) or prevent cognitive impairments in adults with brain tumours.

**Methods:**

We performed a literature search of the Ovid MEDLINE, PsychINFO and PsycTESTS databases from commencement until September 2021.

**Results:**

In total, 9998 articles were identified by the search strategy; an additional 14 articles were identified through other sources. Of these, 35 randomised and nonrandomised studies were deemed to meet the inclusion/exclusion criteria of our review and were subsequently included for evaluation. A range of interventions were associated with positive effects on cognition, including pharmacological agents such as memantine, donepezil, methylphenidate, modafinil, ginkgo biloba and shenqi fuzheng, and nonpharmacological interventions such as general and cognitive rehabilitation, working memory training, Goal Management Training, aerobic exercise, virtual reality training combined with computer‐assisted cognitive rehabilitation, hyperbaric oxygen therapy and semantic strategy training. However, most identified studies had a number of methodological limitations and were judged to be at moderate‐to‐high risk of bias. In addition, it remains unclear whether and to what extent the identified interventions lead to durable cognitive benefits after cessation of the intervention.

**Conclusion:**

The 35 studies identified in this systematic review have indicated potential cognitive benefits for a number of pharmacological and nonpharmacological interventions in patients with brain tumours. Study limitations were identified and further studies should focus on improved study reporting, methods to reduce bias and minimise participant drop‐out and withdrawal where possible, and consider standardisation of methods and interventions across studies. Greater collaboration between centres could result in larger studies with standardised methods and outcome measures, and should be a focus of future research in the field.

## Graphical abstract

This systematic review provides a comprehensive overview of 35 studies of interventions for cognition in adult patients with brain tumours, identifying potential cognitive benefits for a number of pharmacological or nonpharmacological interventions. There were limitations in the identified studies, and further studies should focus on improved study reporting and methods (including blinding of outcome assessors) to reduce bias, methods to minimise participant drop‐out and withdrawal where possible, and consider standardisation of methods and interventions across studies.

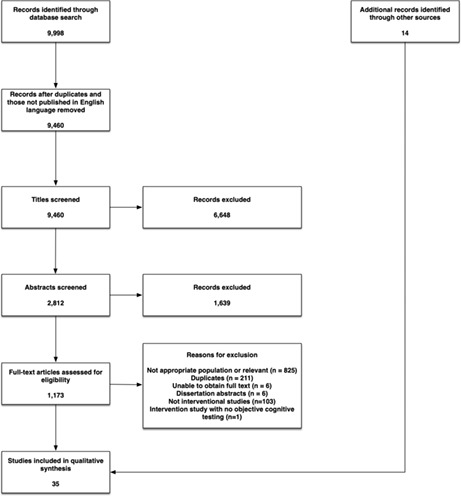

## 1 INTRODUCTION

Impairments in cognition are common in patients with current and previously treated brain tumours,^1^, ^2^ and likely arise from a range of complex, interacting risk factors including patient‐, tumour‐ and treatment‐specific variables. Some of the most well‐studied putative influences on cognitive dysfunction include cranial irradiation, surgery, tumour characteristics including volume and location, and anti‐epileptic drug use.^1^ There is evidence indicating that cognitive impairments negatively influence patient outcomes including quality of life (QOL) and survival.^3^ As such, there is great interest in methods to ameliorate (improve) or prevent cognitive impairments in this population.

To this end, a number of pharmacological and nonpharmacological interventions aiming to ameliorate or prevent cognitive impairment have been evaluated in patients with brain tumours. The pharmacological interventions evaluated include psychostimulants such as modafinil^4^, ^5^ and methylphenidate,^5^, ^6^ as well as the N‐Methyl‐D‐aspartate receptor antagonist memantine^7^ and the reversible cholinesterase inhibitor donepezil.^8^, ^9^ The exact pharmacological mechanisms through which these interventions could exert an effect remain to be elucidated, but could result from their involvement in important neurotransmitter pathways. For example, donepezil, by inhibiting the breakdown of acetylcholine, could prolong and improve cholinergic function, which is associated with learning and memory.^10^

Nonpharmacological interventions that have been used in patients with brain tumours are varied and include cognitive rehabilitation,^11^, ^12^, ^13^, ^14^, ^15^, ^16^, ^17^ mindfulness training,^18^ exercise therapy,^19^ hyperbaric oxygen therapy^20^ and dietary intervention.^21^ Given the wide diversity in nonpharmacological approaches, the potential mechanisms through which these interventions could bring about a change in cognitive function are likely to be equally varied. For example: cognitive rehabilitation may involve brain plasticity through the retraining of cognitive capabilities in domains such as attention and working memory, or involve compensation strategies including memory aids^22^; exercise therapy may bring positive effects through increased cerebral blood flow and hippocampal neurogenesis as well as changes in neurotransmitter release, arousal levels and brain structure including brain derived neurotrophic factor‐associated nerve growth^23^, ^24^; and hyperbaric oxygen therapy has been shown to affect multiple cellular and molecular pathways involving neuroprotection, neuroinflammation, oxidative stress, mitochondrial function, neurogenesis, apoptosis and angiogenesis.^25^

Despite the range of interventions studied, none are routinely and universally applied in the clinical setting. The aim of this systematic review was to provide a comprehensive overview of the interventions used to ameliorate or prevent cognitive dysfunction in adults with current and previously treated brain tumours, critically evaluate the identified studies and the evidence for each intervention, and to discuss the practical implications and future perspectives for the neuro‐oncology community.

## 2 MATERIALS AND METHODS

### 2.1 Study approval

We obtained approval for the protocol of this systematic review from the International Prospective Register of Systematic Reviews (PROSPERO [https://www.crd.york.ac.uk/prospero/]; approval number: CRD42017072976). The search strategy and title and abstract screening process incorporated both interventional and noninterventional studies evaluating the influences on cognitive function and outcomes in patients with brain tumours, but this manuscript focuses on the interventional studies.

### 2.2 Data sources and search strategy

We prespecified the methods used in this systematic review and present them in accordance with the latest Preferred Reporting Items for Systematic Reviews and Meta‐Analyses guidelines.^26^ We performed a literature search of the electronic databases of Ovid MEDLINE(R) (1946 to Present [April 2018]), PsycINFO (1806 to April Week 1 2018) and PsycTESTS (1910 to March 2018). Subsequently, we performed a top‐up search using the same databases (Ovid MEDLINE(R), 1946 to September 24, 2021; PsycINFO, 1806 to September Week 3 2021; PsycTESTS, 1910 to September 2021) incorporating a filter to focus on articles published since 2018. We incorporated Medical Subject Heading terms (Appendix S1) into our search strategy, which combined the three broad content areas of brain tumour, cognition and outcome/plasticity/recovery using the Boolean operator “and” (Appendix S2). In order to identify further potentially relevant literature, we reviewed the reference lists of studies identified by our search strategy. The search strategy was deliberately broad to minimise the risk of missing relevant studies.

### 2.3 Inclusion/exclusion criteria

The inclusion criteria for this systematic review of interventional studies were as follows:

Study design: Randomised and nonrandomised studies published in English language;

Population: Adult brain tumour patients (aged ≥18 years) that underwent objective cognitive function testing;

Intervention: Any intervention type used to ameliorate or prevent cognitive dysfunction;

Outcome: Objective (not self‐reported) measure of cognition.

There was no restriction on the type of intervention considered in this review, with both pharmacological and nonpharmacological interventions included. The primary outcome measure did not have to be cognitive function, as long as cognition was assessed using an objective (not self‐reported) measure.

We excluded review papers, case reports involving a single patient, nonhuman studies, abstracts from dissertations, chapters from or whole textbooks, studies that focussed on children, and studies that did not specifically employ an intervention to address cognitive impairment prevention or amelioration. In addition, we excluded studies where the intervention under investigation was cranial irradiation itself (e.g., the use of hippocampal‐sparing techniques).

### 2.4 Screening

The screening of manuscript titles to identify articles of potential relevance was performed by MAK (a neurosurgeon), after which the abstract screening was performed independently by MAK and BHMH (another neurosurgeon). Full‐text articles were reviewed where ambiguity existed in relation to the inclusion of an article. Disagreements between the two authors were resolved by consensus.

### 2.5 Data extraction

A standardised data extraction proforma was used by MAK and JOE (a postdoctoral research associate in cognitive neuroscience) to extract relevant data from the studies identified as meeting the criteria for inclusion, and the studies were critically appraised. If data to complete the data extraction process were unavailable in the manuscript, authors of the studies were contacted for clarification via email.

### 2.6 Risk of bias assessment

The Cochrane Risk of Bias 2 assessment tool^27^ was used to assess the risk of bias among the randomised studies, whereas for nonrandomised studies the National Heart, Lung and Blood Institute (NHLBI) Quality Assessment Tool for Before‐After (Pre–Post) Studies With No Control Group (available at: https://www.nhlbi.nih.gov/health‐topics/study‐quality‐assessment‐tools) was used. The risk of bias assessments were performed independently by two authors (MAK and JOE), and disagreements were resolved through consensus. Where there was an unclear risk of bias, authors were contacted for clarification via email. Risk‐of‐bias plots were created using the *robvis* tool.^28^ Further information regarding the Risk of Bias assessments is provided in Appendix S3.

## 3 RESULTS

### 3.1 Selected articles

**FIGURE 1:**
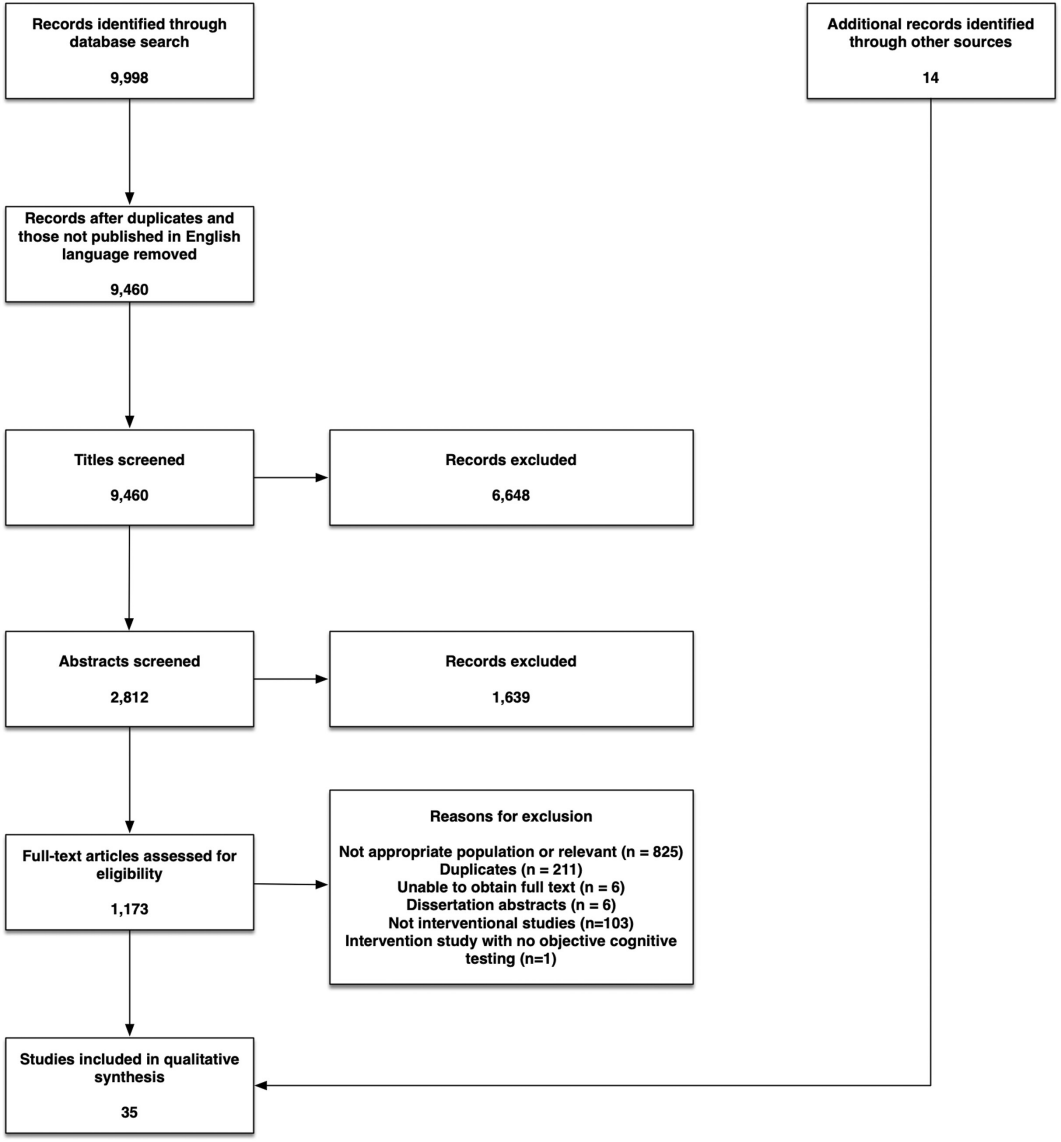
Study flow chart.

We identified 9998 articles using the above‐described search strategy (a flow chart illustrating the selection process is provided in Figure 1). Following the exclusion of duplicate articles and those not published in English, there were 9460 articles remaining for title screening, of which we selected 2812 for abstract review. Full‐text review was performed on 1173 of these articles and an additional 14 articles that were identified through other sources. There were 35 manuscripts that met the inclusion and exclusion criteria of this review.

### 3.2 Characteristics of included studies and study settings

Tables 1 and 2 provide a summary of the 35 included studies.

**TABLE 1:** Basic information on the studies included in this systematic review.

	*N*
Total number of studies	35
Type of study	
Randomised controlled trial	22
Prospective uncontrolled/pilot study	7
Phase II open‐label study	2
Phase IIa single‐arm study	1
Case series report and prospective observational study	1
Nonrandomised controlled study	1
Retrospective case–control study	1
Type of intervention	
Pharmacological	14
Nonpharmacological	21
Location of study authors	
Europe	13
North America	13
More than one continent	4
Asia	4
South America	1
Decade study published	
2020 onwards	7
2010–2019	21
2000–2009	6
1990–1999	1

**TABLE 2:** Summary of study characteristics.

Author	Year	Location of study authors	Sample size	Intervention	Aim of intervention in relation to cognitive dysfunction
Randomised studies					
Boele	2013	Netherlands	37	Modafinil	Prevention
Brown	2013	USA and Canada	554^a^	Memantine	Prevention
Butler	2007	USA	68	d‐threo‐methylphenidate	Prevention
Chen	2019	China	100	Shenqi fuzheng	Prevention
Durà Mata	2018	Spain	84	Cognitive rehabilitation	Prevention
Gehring	2009	Netherlands	140	Cognitive rehabilitation	Prevention
Gehring	2012	Netherlands and USA	34	Methylphenidate/modafinil	Amelioration
Gehring	2020	Netherlands	34	Aerobic exercise	Prevention
Hulshof	2002	Netherlands	7	Hyperbaric oxygen therapy	Amelioration
Kaleita	2006	USA	30	Modafinil	Amelioration
Laigle‐Donadey	2019	France	41	Dexamphetamine	Prevention
Locke	2008	USA	19	Cognitive rehabilitation	Amelioration
Page	2015	USA	54	Armodafinil	Prevention
Peng	2016	China and USA	94	Lidocaine	Prevention
Porter	2022	USA	297	Armodafinil	Prevention
Rapp	2015	USA	198	Donepezil	Amelioration
Richard	2019	Canada	26	Goal Management Training	Amelioration
Taylor	2020	USA and Netherlands	23	Cognitive rehabilitation	Amelioration
Van der Linden	2021	Netherlands	99	Cognitive rehabilitation	Prevention
Voss	2022	Germany	50	Ketogenic diet and intermittent fasting	Prevention
Yang	2014	Korea	38	Virtual reality training combined with computer‐assisted cognitive rehabilitation	Amelioration
Zucchella	2013	Italy	53	Cognitive rehabilitation	Amelioration
Nonrandomised studies					
Attia	2012	USA	34	Ginkgo biloba	Prevention
Braun	2021	USA	20	CogMed Working Memory Training	Amelioration
Han	2015	Korea	55	Cognitive rehabilitation	Prevention
Hassler	2010	Austria	26	Holistic mnemonic training	Prevention
Hojan	2020	Poland	203	Comprehensive rehabilitation	Prevention
Maschio	2015	Italy	16	Cognitive rehabilitation	Prevention
Meyers	1998	USA	44	Methylphenidate	Amelioration
Miotto	2013	Brazil, USA and UK	21	Semantic strategy training	Prevention
Miotto	2014	Brazil	24	Semantic strategy training	Prevention
Sacks‐Zimmerman	2015	USA	3	CogMed Working Memory Training	Amelioration
Schellart	2011	Netherlands	43	Hyperbaric oxygen therapy	Amelioration
Shaw	2006	USA	35	Donepezil	Amelioration
Yu	2019	Korea	143	Intensive rehabilitation therapy	Prevention

^a^ There were 554 patients randomised to this study, but cognitive outcomes at 24 weeks were evaluated in only 280 of these.

### 3.3 Study quality and level of evidence

Of the included studies, the majority were randomised controlled trials (*n* = 22, 62.9%). There were also 7 (20.0%) prospective uncontrolled/pilot studies, and one (2.9%) retrospective case–control study. Three (8.6%) of the identified studies were presented in abstract form, with no full‐text study available at the time this review was conducted.^4^, ^11^, ^14^

### 3.4 Included studies

To facilitate comparison between studies, the 35 studies are grouped here into pharmacological (*n* = 14, 40.0%) and nonpharmacological (*n* = 21, 60.0%) intervention studies.

#### 3.4.1 Pharmacological studies

Of the 14 pharmacological intervention studies identified, 11 were randomised^4^, ^5^, ^6^, ^7^, ^8^, ^29^, ^30^, ^31^, ^32^, ^33^, ^34^ and three were nonrandomised.^9^, ^35^, ^36^ The pharmacological interventions evaluated included the central nervous system (CNS) stimulants modafinil,^4^, ^5^, ^29^ d‐threo‐methylphenidate HCl (d‐MPH),^6^ methylphenidate,^5^, ^36^ dexamphetamine^30^ and armodafinil,^31^, ^33^ the local anaesthetic agent lidocaine,^32^ the cholinesterase inhibitor donepezil,^8^, ^9^ the N‐Methyl‐D‐aspartate receptor antagonist memantine,^7^ and the herbs gingko bilboa^35^ and shenqi fuzheng.^34^ The comparator arm was placebo in eight of the 11 randomised pharmacological intervention studies^6^, ^7^, ^8^, ^29^, ^30^, ^31^, ^32^, ^33^; in the three remaining studies, there was no placebo and the comparators were either methylphenidate and modafinil,^5^ two different doses of modafinil,^4^ or there was no comparator at all.^34^ In three studies, pre‐existing subjective or objective cognitive impairment was a criterion for inclusion in the study^4^, ^5^, ^36^; hence, these studies were cognitive deficit amelioration (as opposed to prevention) studies. A further two studies evaluated the use of donepezil in patients who received cranial irradiation at least 6 months prior,^8^, ^9^ and can thus also be considered amelioration studies.

Study sample sizes ranged from 30^4^ to 297.^33^ Seven were multicentre,^6^, ^7^, ^8^, ^29^, ^30^, ^31^, ^33^ and seven were single‐centre studies.^4^, ^5^, ^9^, ^32^, ^34^, ^35^, ^36^ Participant ages were broadly similar across all studies, with average ages across all studies in the fifth or sixth decade of life. Gender data were available for all studies, and most studies were relatively well‐balanced. Ethnicity data were available for eight of the studies.^6^, ^7^, ^8^, ^9^, ^31^, ^32^, ^33^, ^35^ Loss to follow‐up and study withdrawal was common. Indeed, accrual to the Butler et al.^6^ study was slower than anticipated, resulting in premature closure of the study due to withdrawal of support from the sponsoring drug company. Adverse effects of the interventions were reported in most studies.

Most studies included patients with two or more pathologies, often gliomas combined with other pathologies, including meningiomas,^4^, ^9^, ^29^, ^30^, ^31^, ^32^ metastatic brain tumours,^6^, ^8^ CNS lymphoma^4^, ^30^ and medulloblastoma.^9^, ^30^, ^36^ Two studies included only patients with brain metastases.^7^, ^34^ One study reported including patients undergoing prophylactic cranial irradiation.^8^ Included patients had received varying treatments including surgery, radiotherapy and/or chemotherapy. One study measured cognition using a single general cognitive screening tool (Mini‐Mental State Examination^6^), whereas most of the remaining studies used a more comprehensive battery of neuropsychological tests (although there was much variability in the specific tests used between studies). Timing of post‐intervention assessments varied, with follow‐up assessments continued to 4 weeks^31^ or 6 months^34^ after radiotherapy; 30 days,^5^ 8 weeks,^33^ 12 weeks/3 months,^4^, ^6^, ^29^, ^30^ 24 weeks,^8^ 30 weeks,^9^, ^35^ or 6 months after surgery^32^; or 52 weeks after commencing the study drug.^7^ In one study, the timing of cognitive testing post‐baseline was not explicitly specified.^36^ In six studies, outcome assessments were performed by individuals blind to the participants' treatment^7^, ^8^, ^29^, ^30^, ^31^, ^33^; in the remainder, either no blinding of outcome assessments was performed or no information was provided.

#### 3.4.2 Nonpharmacological studies

Of the 21 nonpharmacological studies identified, 11 were randomised^11^, ^12^, ^13^, ^14^, ^15^, ^18^, ^19^, ^21^, ^37^, ^38^, ^39^ and 10 were nonrandomised.^16^, ^17^, ^20^, ^22^, ^40^, ^41^, ^42^, ^43^, ^44^, ^45^ Most of the nonpharmacological studies evaluated cognitive training or rehabilitation programmes.^11^, ^12^, ^13^, ^14^, ^15^, ^16^, ^17^, ^18^, ^22^, ^38^, ^39^, ^40^, ^41^, ^43^, ^44^ The cognitive rehabilitation was delivered through a range of modalities, including computer programs and virtual reality. Other interventions studied include aerobic exercise,^19^ hyperbaric oxygen,^20^, ^37^ ketogenic diet with intermittent fasting^21^ and broad multidisciplinary rehabilitation.^42^, ^45^ Due to the nature of the nonpharmacological interventions, a placebo intervention was not described in studies. Comparator groups comprised of care‐as‐usual,^11^, ^13^, ^15^ wait‐list control group,^12^, ^18^, ^38^ delayed hyperbaric oxygen treatment,^37^ active control,^14^, ^18^, ^19^, ^39^ standard diet,^21^ healthy controls^20^, ^43^ and stroke patients receiving the same rehabilitation intervention.^16^, ^45^ Six nonrandomised studies did not include a comparator group. In nine of the 21 nonpharmacological intervention studies, pre‐existing subjective or objective cognitive impairment was a criterion for inclusion in the study^13^, ^14^, ^15^, ^18^, ^20^, ^37^, ^39^, ^40^, ^44^; hence, these studies evaluated cognitive deficit amelioration (as opposed to prevention).

The sample sizes of the nonpharmacological studies included in this review ranged from 3^44^ to 143.^45^ Five of the 21 studies were multicentre,^12^, ^19^, ^21^, ^38^, ^42^ and the remainder were single‐centre studies.^11^, ^13^, ^14^, ^15^, ^16^, ^17^, ^18^, ^20^, ^22^, ^37^, ^39^, ^40^, ^41^, ^43^, ^44^, ^45^ Like the pharmacological intervention studies, the mean/median age of participants in the nonpharmacological intervention studies was similar, in the fifth and sixth decades of life, although participant ages were not reported in one study.^11^ Gender of participants was not reported in two of the studies,^11^, ^21^ but where it was, there was reasonable gender balance in most studies. Ethnicity data were available for six of the studies.^20^, ^22^, ^40^, ^41^, ^43^, ^45^ Like the pharmacological intervention studies, loss to follow‐up and study withdrawal was common in the included studies.

Some of the included nonpharmacological studies recruited solely glioma patients,^11^, ^12^, ^14^, ^19^, ^21^, ^41^, ^43^, ^44^ and others included multiple pathologies including ependymoma,^37^, ^42^ subependymoma,^42^ medulloblastoma,^37^ neuroblastoma,^37^ meningioma,^13^, ^15^, ^17^, ^18^, ^20^, ^22^, ^38^, ^39^, ^42^ craniopharyngioma,^40^ metastasis^17^, ^20^, ^39^ and non‐Hodgkin lymphoma.^20^ Like the studies evaluating pharmacological interventions, those evaluating nonpharmacological intervention included patients that had received varying treatments including surgery, radiotherapy and/or chemotherapy. Although many of the studies used a combination of several cognitive tests spanning multiple cognitive domains, some studies relied on the use of a single cognitive measure such as the MMSE,^39^ Addenbrooke's Cognitive Examination III,^42^ or Repeatable Battery for the Assessment of Neuropsychological Status (R‐BANS)^13^; in the latter study, the R‐BANS was not completed by most patients at follow‐up, and thus, no longer‐term follow‐up data were provided. One study, published in abstract form, did not describe the specific cognitive tests used.^11^ Like the pharmacological intervention studies, timing of cognitive assessments post‐intervention varied for the nonpharmacological intervention studies. Studies continued assessments to 4weeks post‐baseline^15^ or at the end of the four‐week rehabilitation,^16^ 1 month after radiation therapy^21^ or rehabilitation treatment,^39^ more than 1 month after admission,^45^ 3 months^13^, ^44^ (although cognitive assessment data were not provided at follow‐up in the Locke et al. study [see “Risk of bias” section below]), 4 months post‐intervention,^18^, ^20^ 12 weeks,^41^, ^42^ 6 months,^12^, ^14^, ^17^, ^19^, ^37^, ^40^ 7 months after surgery^11^ and 1 year post‐surgery.^38^ In eight studies, outcome assessments were performed by individuals blind to the participants' treatment^12^, ^15^, ^16^, ^18^, ^22^, ^38^, ^41^, ^43^; in the remainder, either no blinding of outcome assessments was performed or no information was provided.

### 3.5 Risk of bias

**FIGURE 2:**
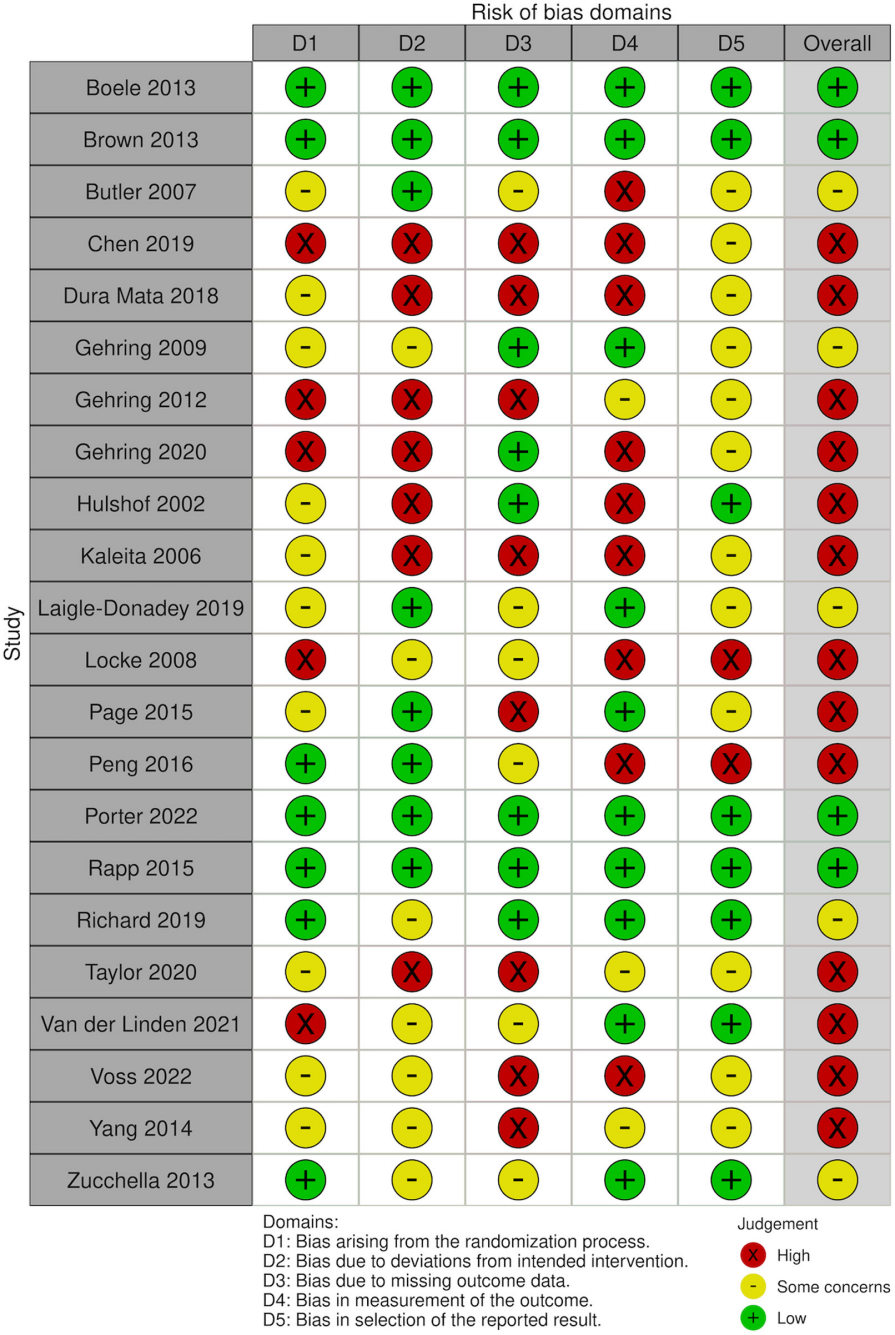
Graphic showing the results of the risk of bias assessment of the randomised studies in our review, ordered by the surname of the first author. The risk of bias for randomised studies was performed using the Cochrane Risk of Bias assessment tool.

**FIGURE 3:**
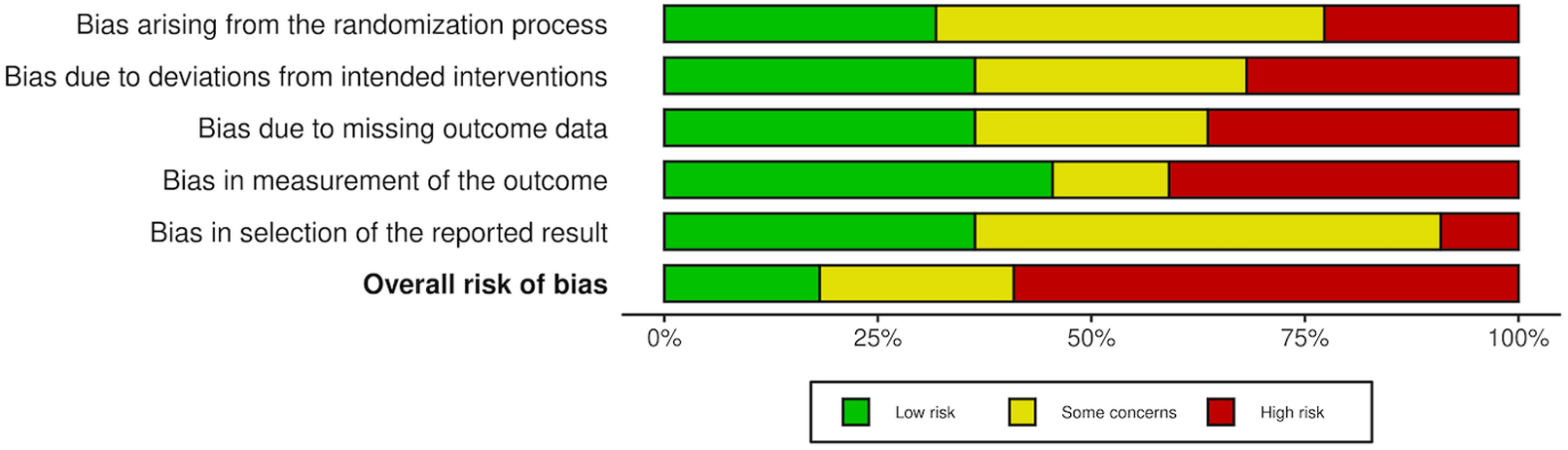
Graphic showing the results of the risk of bias assessment of the randomised studies in our review, separated by specific Cochrane Risk of Bias domain.

**FIGURE 4:**
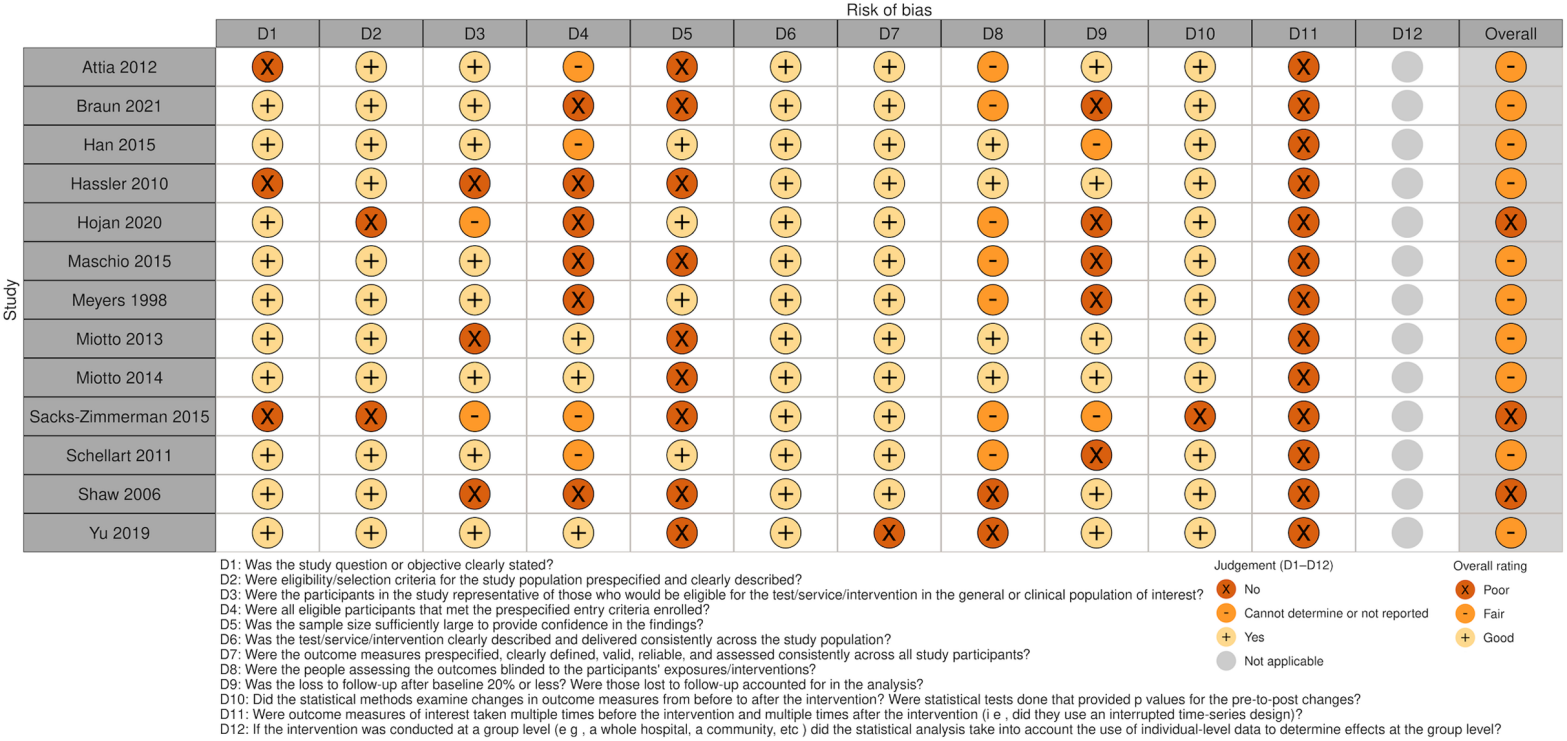
Graphic showing the results of the risk of bias assessment of the nonrandomised studies in our review, ordered by the surname of the first author. The risk of bias for nonrandomised studies was performed using the National Heart, Lung and Blood Institute (NHLBI) Quality Assessment Tool for Before‐After (Pre–Post) Studies With No Control Group.

**FIGURE 5:**
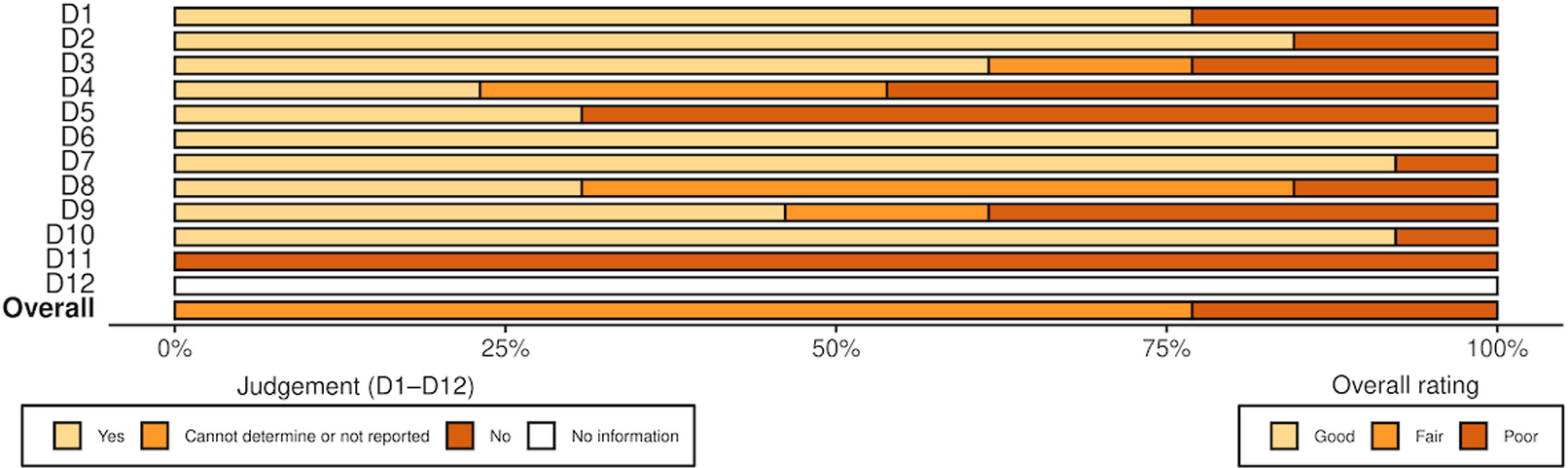
Graphic showing the results of the risk of bias assessment of the nonrandomised studies in our review, separated by specific question from the National Heart, Lung and Blood Institute (NHLBI) Quality Assessment Tool for Before–After (Pre–Post) Studies With No Control Group.

The detailed risk of bias assessment results are shown in Appendix S3 and in Figures 2, 3, 4, 5, which show that many of the included studies were at high risk of bias. Of the 22 randomised studies evaluated using the Cochrane Risk of Bias 2 tool, only four were found to have a low risk of bias overall (Figure 2).^7^, ^8^, ^29^, ^33^ Of the 13 nonrandomised studies included in this systematic review, three were assessed as having an overall rating of “poor” using the NHLBI Quality Assessment Tool for Before‐After (Pre–Post) Studies With No Control Group,^20^, ^42^, ^44^ and the remainder were assessed as having an overall rating of “fair” (Figure 4)^9^, ^16^, ^17^, ^22^, ^35^, ^36^, ^40^, ^41^, ^43^, ^45^; thus, none of the included studies were evaluated as “good” overall.

### 3.6 Effects of interventions

The study interventions, comparisons, outcome measures and reporting were too heterogenous to pool data. Thus, the results of included studies are reported separately. A summary of the studies is provided here. Effect sizes are provided where reported.

#### 3.6.1 Pharmacological studies

Attia et al.^35^ evaluated the effect of 120 mg ginkgo biloba administered daily for 24 weeks, followed by a washout period of 6 weeks, on cognitive function, QOL and mood in irradiated brain tumour patients recruited from a US centre. There was no randomisation of participants, but participants served as their own control in this open‐label study. The specific brain tumour subtypes and number of patients that received chemotherapy were not specified, but all participants had received partial or whole‐brain radiotherapy at least 6 months prior to enrolment, had no imaging evidence of tumour progression in the preceding 3 months, were on stable or reducing doses of steroid therapy, and had no brain tumour treatment planned during the course of the study. Rates of steroid and anti‐epileptic drug use were not reported. Of the 34 participants enrolled, 19 (55.9%) completed 24 weeks of treatment. Cognition was evaluated using a battery of tests covering global cognitive functioning, attention and concentration, visuoconstructional skills, verbal fluency, executive function, verbal learning and memory, and figural memory. Following 24 weeks of treatment, time taken to complete the Trial Making Test A (TMT‐A) had significantly decreased (*p* = 0.002). Trail Making Test B (TMT‐B) performance also improved significantly from 24 to 30 weeks, but TMT‐A performance did not. Immediate and delayed recall scores on the Modified Rey‐Osterrieth Figure also significantly improved (*p* < 0.001 and *p* = 0.002, respectively). There were no significant changes identified in measures of global cognitive function (MMSE), verbal fluency (F‐A‐S Test), or attention/concentration and working memory (Digit Span Test Total).

Boele et al.^29^ investigated the effects of modafinil on fatigue, health‐related QOL, depression and cognitive outcomes in a double‐blind, placebo‐controlled randomised study of 37 patients with primary brain tumours recruited from three Dutch institutions. The tumour types of participants included meningioma (32.4%), low‐grade glioma (37.8%) and high‐grade glioma (29.7%). Prior radiotherapy and chemotherapy were received in 43.2% and 21.6% of participants at some point, respectively, and although a criterion for study inclusion was no evidence of tumour recurrence in the preceding 6 months, 5.4% of participants experienced disease progression during the study intervention. No data on steroid use of participants were provided, but 54.1% of participants were receiving anti‐epileptic therapy. Modafinil was administered as a 200 mg per day dose, increased to 400 mg after 1 week and, following a washout period of 1 week, the opposite treatment was initiated (i.e., those receiving modafinil then received placebo). Cognition was assessed using tests covering verbal memory, working memory, attentional functioning, information processing, executive functioning and psychomotor speed, and six cognitive domains were formed based on a principal component analysis. Scores were found to not differ between the experimental conditions. Compared to baseline, patients improved after treatment with both modafinil and placebo in the domains of working memory (*p* = 0.040 and 0.043, respectively) and information processing (*p* = 0.036 and 0.040, respectively). Attentional functioning scores were also noted to significantly improve after placebo (*p* = 0.015) and modafinil treatment (*p* = 0.013) compared with baseline.

Brown et al.^7^ evaluated the effects of memantine, which acts on the glutamatergic system as an N‐methyl‐D‐aspartate receptor antagonist, in a large double‐blind, placebo‐controlled study of 554 randomised patients with brain metastases recruited from 143 centres in the United States and Canada. Stable systemic disease in the 3 months preceding study entry was required for participation in the study. The primary tumour sites of the 508 randomised patients that were eligible for study participation included lung (69.9%), breast (14.8%), colon (1.0%) and other (14.4%). Prior chemotherapy was administered in 44.7% of participants, and 65.0% and 26.6% were receiving steroids and whole‐brain radiotherapy at study entry, respectively. Rates of anti‐epileptic medication use were not reported. Participants were randomised to receive either placebo or memantine (20 mg daily) within 3 days of initiating radiotherapy and continuing for 24 weeks. Imputation was conducted for participants with missing assessments who had not withdrawn from the study as a result of death. Cognitive tests administered included tests of memory (Hopkins Verbal Learning Test‐Revised [HVLT‐R]), processing speed (TMT‐A), executive function (TMT‐B), verbal fluency (Controlled Oral Word Association [COWA]) and global cognitive function (MMSE). Mean cognitive decline was reported for 280 participants at 8, 16 and 24 weeks. The primary endpoint was Hopkins Verbal Learning Test‐Revised (HVLT‐R) at 24 weeks. There was less decline in delayed recall in the intervention arm of the study at 24 weeks, but the difference between groups did not reach statistical significance (*p* = 0.059); this was attributed to attrition. A secondary endpoint, time to cognitive decline, was significantly longer in the memantine arm (hazard ratio [HR] 0.78, 95% confidence interval (CI) 0.62–0.99, *p* = 0.01); the probability of cognitive function failure at 24 weeks was 53.8% in the memantine arm and 64.9% in the placebo arm. A cognitive functioning composite score was also calculated, and the median change was −0.41 (interquartile range −1.30 to 0.12) in the control group and −0.03 (−0.90 to 0.72) in the intervention group at 24 weeks (*p* = 0.02). This indicated a stability of cognitive function in the intervention group and a decline in the control group. The most common adverse events were fatigue, alopecia, nausea and headache, but no difference was observed in the adverse events reported between groups (risk ratio 1.00; 95% CI 0.76–1.32). A notable study finding was that more memantine group participants were receiving steroids at study entry than the control group (*p* = 0.05); this could indicate more symptoms and mass effect from brain metastases in the memantine group, and thus could have led to a worse cognitive outcome at this time point, although the difference in steroid use was not maintained over time.

Butler et al.^6^ evaluated the effect of d‐MPH compared to placebo on fatigue, QOL and cognition in patients with metastatic or primary brain tumours undergoing partial or whole‐brain radiotherapy. Sixty‐eight patients were randomised (34 in each arm) to this double‐blind phase III US study, of whom 49% had primary brain tumours and the remainder had metastatic brain tumours; details on specific subtypes were not provided. Twenty‐five percent of participants received chemotherapy, but the number of participants on steroid or anti‐epileptic therapy was not provided. The study terminated early due to low accrual. A single measure of global cognitive function was administered (MMSE). No significant difference in global cognition was observed between the two arms at 8 weeks following treatment or indeed across the entire follow‐up period that spanned up to 12 weeks post‐radiotherapy.

Chen et al.^34^ compared the daily injection of 250 mL shenqi fuzheng, a Chinese traditional herb medicine, for 4 weeks combined with radiotherapy to radiotherapy alone in 100 patients with brain metastases recruited from a single centre in China; blinding of participants was therefore not possible. All patients had brain metastases from lung primary disease, and the underlying primary pathology was adenocarcinoma in 80%, squamous cell carcinoma in 5%, and small cell lung cancer in 15%. Participant withdrawals and chemotherapy, steroid therapy, or anti‐epileptic medication use were not described in the published manuscript. Results were reported at baseline and 3, 6, 9 and 12 months after commencing radiotherapy for 100 participants; 58 in the control group and 48 in the shenqi fuzheng injection group, with no participants reported as being lost to follow‐up. The study presented the results of the neurocognitive testing, which was obtained through administration of the MMSE and Montreal Cognitive Assessment (MoCA), in graphical form with no numerical or statistical data presented in text form in either the manuscript text or tables. The authors described that total MMSE scores declined over time, principally in scores of memory ability and verbal ability. Memory ability, as assessed through the MoCA, was described as dropping significantly, reaching the lowest point at 6 months and then levelling off. Slight declines were reported in executive capabilities and orientation forces, whereas naming, calculative, verbal and orientation abilities did not show any significant changes over the period of study. Total MoCA scores were also reported to decline and reached their lowest point at 6 months. Participants in the intervention group were reported as having less of a decline in scores following radiotherapy. The radiotherapy was described as primarily affecting memory ability. No adverse events were reported in the study's manuscript.

Gehring et al.,^5^ in a randomised, open‐label trial, compared the effects of two CNS stimulants on cognitive function and symptoms in 34 randomised primary brain tumour patients recruited from 11 Dutch hospitals through three treatment arms: immediate‐release methylphenidate, sustained‐release methylphenidate and modafinil. Most (88%) of the participants had a World Health Organization grade II–IV glioma, with the remainder including medulloblastoma (4%), primary CNS lymphoma (4%) and hemangiopericytoma (4%). A history of chemotherapy and radiotherapy treatment was confirmed in 88% and 83%, respectively, but data on steroid and anti‐epileptic use were not provided. An inclusion criterion of the study was the subjective complaint of cognitive decline or fatigue by participants. Patients with tumour progression were excluded. A battery of cognitive tests, including measures of attention, processing speed, memory and executive function, was reported for 24 of the 34 randomised participants up to a median of 30 days after treatment. The two methylphenidate arms (immediate‐ and sustained‐release methylphenidate) were combined during analysis of the study data owing to low participant accrual. Improvement in Digit Symbol and TMT‐B scores and deterioration in COWA and HVLT‐R Delayed Recognition at the group level were observed after stimulant treatment, with the deterioration not associated with radiographic evidence of disease progression. Group scores differed in Digit Span and TMT‐A according to stimulant group. At the individual level, 32% of patients improved in TMT‐B scores following stimulant treatment, and this was significant according to the binomial test and was corrected for practice effects; however, no statistically significant rate of improvement was found for any of the other cognitive tests. Furthermore, the proportion of patients with score improvements did not differ between the two stimulant groups. It was also noted that patients with lower baseline scores experienced a greater improvement in TMT‐B scores only (*p* < 0.001).

Kaleita et al.,^4^ published as a conference abstract only, compared the cognitive effects of two different doses of modafinil (200 or 400 mg/day) in 30 patients with brain tumours in a double‐blind dose‐controlled randomised US study. Participants received the assigned treatment for 3 weeks, followed by a 1‐week washout period and an 8‐week open‐label extension. The tumour types of included participants comprised of glioblastoma multiforme (27%), anaplastic glioma (33%), low‐grade glioma (33%), meningioma (3%) and CNS lymphoma (3%). A history of radiotherapy and chemotherapy was confirmed in 87% and 70% of participants, respectively. Rates of steroid and anti‐epileptic use in participants, as well as disease stability prior to enrolment, were not provided. A cognitive test battery covering the domains of executive function, attention and decision‐making, and verbal fluency was administered to 30 participants at baseline, and following eight and 12 weeks of drug use. The authors noted, compared to baseline, a significant improvement at 12 weeks across all cognitive tests: TMT‐A (*p* = 0.002), TMT‐B (*p* < 0.0001), Verbal Fluency (*p* = 0.002) and Symbol Digit Modalities‐Oral (*p* = 0.006) and ‐Manual (*p* = 0.004). In addition, significant differences were noted at 8 weeks for all tests.

Laigle‐Donadey et al.,^30^ in a double‐blind randomised trial, assessed the efficacy and tolerability of 30 mg/day dexamphetamine compared to placebo in 46 primary brain tumour patients complaining of severe fatigue. Participants were recruited from 7 centres in France and included tumour types comprised of WHO grade IV glioma (41.5%), WHO grade III glioma (34.1%), WHO grade II glioma (7.3%), CNS lymphoma (9.8%) and medulloblastoma (7.3%). None of the patients had evidence of progressive disease at enrolment, and 90.2% and 92.7% had been previously treated with radiotherapy and chemotherapy, respectively. The proportion of participants receiving steroid or anti‐epileptic therapy was not documented. Of the 46 enrolled patients, 41 (22 in the intervention arm and 19 in the control arm) underwent a cognitive test battery at baseline and 3 months, which comprised tests of global cognitive function, processing speed, verbal fluency, executive function, attention, concentration, visual‐motor speed, verbal learning and episodic memory. No significant difference was identified in any of the neurocognitive parameters.

Meyers et al.,^36^ in a nonrandomised study, evaluated whether methylphenidate would improve neurobehavioural function in primary brain tumour patients recruited from a single US centre. Patients had either anaplastic glioma (77%), glioblastoma (20%), or medulloblastoma (3%). Although the proportion of patients on chemotherapy, steroid therapy, or anti‐epileptic drugs was not specified, 93% had received prior radiotherapy, and 37% were on “active treatment” (undefined by the authors). A dose escalation study was performed, commencing at 10 mg daily, increased by 10 mg daily every 2 weeks until a response was achieved or there was dose‐limiting toxicity. There was no control group. Cognition was evaluated at baseline and on‐treatment (timing not specified) through a battery of tests evaluating the domains of attention, graphomotor speed, memory, verbal fluency, visuo‐motor scanning speed and executive function. Data were available for 30 of the 44 patients enrolled. Mean test scores indicated significant improvements in memory, executive function, visual‐motor function, psychomotor speed, and motor speed and dexterity. There was no relationship identified between mood and cognition.

Page et al.^31^ evaluated the role of armodafinil in reducing fatigue in 54 primary brain tumour patients undergoing radiotherapy in a double‐blind, placebo‐controlled randomised phase II study, with secondary outcomes including toxicity/adverse events, QOL and cognition. Twenty‐six patients recruited from multiple community and academic institutions in the United States were randomised to receive armodafinil 150 mg daily and 28 to placebo during radiotherapy and for an addition 4 weeks after. Tumour histology in the participants included glioblastoma (61.1%), meningioma (13.0%) and others (26.0%). At baseline, 20.4% of participants had received chemotherapy, 55.6% had received steroid therapy, and 53.7% had received anti‐epileptic drugs. Cognition was evaluated at baseline, end of radiotherapy, and 4 weeks post‐radiotherapy using a battery of tests: the Verbal Fluency‐Category (VF‐C) (Animals), HVLT‐R, TMT‐A, TMT‐B and Digit Span Test‐Backwards. No significant differences were observed between treatment arms at the end of radiotherapy or 4 weeks later in any of the cognitive tests. Armodafinil also did not improve neurocognitive outcomes in the subsets of participants with high or low levels of fatigue at baseline.

Peng et al.,^32^ in a double‐blind, randomised controlled trial, evaluated the effect of lidocaine administration on neuropsychological‐cognitive decline following surgery in patients undergoing supratentorial tumour surgery at a single Chinese hospital. Patients were randomised to receive either 1.5 mg/kg intravenous bolus of lidocaine followed by a 2 mg/kg/h infusion in 0.9% saline until the end of surgery, or a bolus followed by 0.9% saline infusion. Of the 94 patients randomised, 80 were analysed (40 in each arm). Of these 80, 26.3% had WHO grade I–II gliomas, 20% had WHO grade III–IV gliomas, 43.8% had meningiomas, and 10% had other (unspecified) tumour types. Data on radiotherapy, chemotherapy, and anti‐epileptic drug use were only provided for the postoperative period, and the rates were 18.8%, 21.3% and 31.3%, respectively. Data on steroid use were not provided. Cognition was evaluated using the MMSE and Information‐Memory‐Concentration test before surgery and up to 6 months after surgery. The incidence of cognitive decline postoperatively was not significantly different between the two groups.

Porter et al.,^33^ in a phase III double‐blind randomised controlled trial, compared the efficacy in treating fatigue of two armodafinil doses (150 mg or 250 mg daily) to placebo administered for 8 weeks in 297 patients with clinically stable high‐grade (WHO grade III or IV) glioma. Participants were recruited from a total of 365 study sites in the United States. 1–24 months after completing radiotherapy. Prior chemotherapy and corticosteroid use was reported in 80.8% and 39.7% of participants, respectively, but rates of anti‐epileptic medication use were not documented. Cognition at 8 weeks was a secondary objective. Patients were assessed using three cognitive measures (Symbol Digit Modalities Test, COWA and TMT) at baseline and at 4 and 8 weeks of treatment. There were no statistically significant differences identified.

Rapp et al.^8^ compared donepezil (initially 5 mg daily for 6 weeks, subsequently escalated if tolerated to 10 mg daily for 18 weeks) with placebo in a phase III double‐blind randomised controlled trial of 198 irradiated brain tumour survivors recruited from 26 US sites. Most patients (65.7%) had a primary brain tumour (high‐ and low‐grade glioma, meningioma, or other), and the remainder had either brain metastasis (26.8%) or received prophylactic cranial irradiation (7.6%). Rates of chemotherapy, steroid, or anti‐epileptic medication use were not reported. A lack of imaging evidence of disease progression in the 3 months preceding enrolment was an inclusion criterion for the study. Cognitive functioning was assessed using a battery of tests evaluating verbal learning and memory, visuomotor skills, immediate and delayed figure recall, attention, executive function, verbal fluency, and concentration and working memory. Cognitive composite scores (the primary outcome) did not differ significantly between groups at the end of the study (*p* = 0.48), indicating the lack of an overall benefit of treatment on cognition. Significant group differences favouring donepezil were observed for recognition memory (HVLT‐R discrimination, *p* = 0.007; HVLT‐R recognition, *p* = 0.027). The benefits of donepezil were greater for those who were more cognitively impaired prior to study treatment.

Shaw et al.,^9^ in a prospective, open‐label phase II study, evaluated whether donepezil would improve cognitive function, QOL and mood in irradiated brain tumour patients recruited from a single US centre. Patients had primary brain tumours, mostly low‐grade gliomas, but the proportions of specific subtypes were not provided for the 24 of the 35 enrolled participants that were finally analysed. Donepezil 5 mg daily was administered to participants for 6 weeks, which was increased to 10 mg daily for 18 weeks if tolerated followed by a six‐week washout period. Participants served as their own controls. Chemotherapy, steroid, or anti‐epileptic medication use were not reported. Only patients without imaging evidence of disease progression in the 3 months preceding enrolment were eligible for the study. Participants were evaluated using a battery of tests evaluating global cognition function, attention and concentration, visual‐constructional skills, verbal fluency, executive function, verbal memory and figural memory at baseline, 12, 24 and 30 weeks. After 24 weeks of treatment with donepezil, there were significant improvements in test scores across several cognitive domains including attention/concentration, figural memory and verbal memory, as well as a trend toward significance for verbal fluency. However, no significant change was found for global cognitive function or executive function.

#### 3.6.2 Nonpharmacological studies

Braun et al.,^40^ in a nonrandomised single‐arm phase IIa proof‐of‐concept study, aimed to evaluate the effects of CogMed Working Memory Training in high‐ and low‐grade glioma patients recruited from a single US centre. Sixteen participants underwent 5 weeks of 50 min of online training 5 days per week. Tumour types included in the study were described as oligodendroglioma (45%), glioblastoma (30%), astrocytoma (15%), craniopharyngioma (5%) and low‐grade glioma. Half of participants were not on active treatment at baseline, and 85% had no history of disease progression at the time of enrolment. Radiotherapy and chemotherapy were previously administered to 90% and 95% of participants, respectively, but data on steroid or anti‐epileptic use were not provided. A battery of cognitive tests, including tests of working memory screening, auditory/visual working memory, processing speed and verbal and visual delayed memory, was performed at baseline, 3–4 weeks following completion of at‐home training, and 3 and 6 months later. Medium to large effects and significant increases on Wechsler Adult Intelligence Scale (WAIS) Digit Span (ηp2 = 0.35, *p* = 0.01) and WMS Symbol Span (ηp2 = 0.25, *p* = 0.04) were observed from baseline to post‐training; in addition, a small effect and no significant change on R‐BANS Digit Span (ηp2 = 0.05, *p* = 0.40), and medium effects but no statistically significant increases on TMT‐B (ηp2 = 0.20, *p* = 0.07) and R‐BANS‐DM (ηp2 = 0.16, *p* = 0.12) were noted.

Durà Mata et al.,^11^ in a double‐blind, randomised controlled trial published as a conference abstract, evaluated a neurocognitive telerehabilitation program on both cognition and QOL in patients with gliomas undergoing surgery at a single Spanish centre. Eighty‐four patients were randomised to either Neuropersonal trainer, comprising of 60‐min sessions for 12 weeks, or usual treatment. The specific glioma types, chemotherapy, radiotherapy, steroid therapy, anti‐epileptic drug use, and the cognitive tests and analyses performed were not described in the abstract, but the authors reported that the intervention group experienced significant improvements in cognition at 4 and 7 months (*p* = 0.017 and 0.027, respectively) not detected in the control group.

Gehring et al.^12^ evaluated the effects of a cognitive rehabilitation program on both objective and subjective cognitive measures in 140 patients with WHO grade II–III gliomas recruited from 11 Dutch hospitals. Patients were randomised to six weekly individual cognitive rehabilitation sessions each lasting 2 h or wait‐list control. The specific tumour subtypes included astrocytoma (47.9%), oligodendroglioma (32.1%), oligoastrocytoma (14.3%) and presumed glioma (5.7%). A history of radiotherapy and chemotherapy was reported in 61.4% and 10.7% of participants, respectively, and 83.6% received anti‐epileptic medications. Use of steroids was not reported. Patients were required to have no evidence of disease progression for at least 6 months prior to study enrolment. A battery of validated cognitive tests was used to evaluate attention, verbal memory and executive functions at baseline, end of the cognitive rehabilitation program and six‐month follow‐up. There were significant group differences observed over time across measures of attention (*p* = 0.028) and verbal memory (*p* = 0.015), but not executive function. Immediately post‐treatment, no statistically significant group differences in attention or verbal memory scores were observed, but at six‐month follow‐up, a significant group difference was identified for the combined tests of attention (*p* = 0.004) and of verbal memory (*p* = 0.009); effect sizes (*d*) ranged from 0.23 to 0.55.

In another randomised study by Gehring et al.,^19^ 34 patients with WHO grades II–III glioma recruited from 3 Dutch hospitals were randomised to receive either an individualised, home‐based, remotely coached exercise intervention of three aerobic training sessions weekly for 6 months or an active control comprising of a motivational brochure and bimonthly phone calls enquiring about help. Tumour subtypes included in the study were astrocytoma (34.4%), oligodendroglioma (53.1%) and oligoastrocytoma (12.5%). Radiotherapy, chemotherapy and anti‐epileptic drug use was reported in 53.1%, 37.5% and 56.3% of participants, respectively. Imaging stability of the tumour for at least 6 months prior to study entry was an inclusion criterion for the study, whereas the use of corticosteroids (as well as surgery, radiotherapy and chemotherapy) within 6 months prior to study entry was exclusion criteria. Validated cognitive measures were used to evaluate attention, memory and executive function at baseline and after 6 months. Participants in the exercise group were found to have small‐ to medium‐sized better follow‐up scores (effect sizes calculated through linear regression analyses) than those in the control group across several measures of attention and executive function, information processing speed and verbal memory, whereas participants in the control group had slightly better scores on a measure of sustained selective attention.

Han et al.^16^ evaluated for functional improvement in 29 brain tumour patients following four‐week conventional rehabilitation therapy compared to 26 subacute stroke patients in a noncontrolled study that recruited from a single Korean centre. Although the specific tumour subtypes were not reported, 41.4% of the tumour group had benign tumours and 58.6% had malignant tumours. Recurrence was identified in 34.5% of the tumour patients. Rates of radiotherapy, chemotherapy, steroid therapy and anti‐epileptic medication use were not reported. Patients were evaluated in multiple cognitive domains using computerised neuropsychological test at baseline, and global cognitive function assessed using the Korean version of the MMSE at baseline as well as following 4 weeks of rehabilitation. All patients improved in global cognitive function at 4 weeks.

Hassler et al.^41^ evaluated the effect of neurocognitive training on verbal skills, memory skills and attention in a nonrandomised study of 11 patients with high‐grade glioma recruited from a single Austrian centre. No control group was included. Most (63.6%) of the participants had glioblastoma, while 18.2% had anaplastic astrocytoma, 9.1% had anaplastic oligoastrocytoma, and 9.1% had WHO grade III oligodendroglioma. All participants had received chemotherapy and radiotherapy, and were receiving anti‐epileptic medications. Rates of corticosteroid use were not reported. The training comprised of ten weekly 90‐min sessions focusing on holistic mnemonic training. Assessment comprised of a cognitive test battery incorporating measures of verbal memory, visual‐motor speed and executive function, and verbal fluency performed at baseline and 12 weeks later. Comparison of mean baseline and post‐training group differences revealed nonsignificant improvements in all but one cognitive variable (HVLT Total Learning, mean difference = 4.0, *p* = 0.04).

Hojan et al.,^42^ in a prospective observational study evaluated the benefits of 12 weeks of comprehensive inpatient (*n* = 28) and outpatient (*n* = 26) rehabilitation in brain tumour patients recruited from an inpatient and an outpatient facility in Poland. The tumour types included in the study were diffuse astrocytic and oligodendroglial tumours (38.9%), other astrocytic tumours (13.0%), ependymal tumours (7.4%) and meningiomas (40.7%). A history of radiotherapy and chemotherapy was noted in 46.3% and 24.1% of participants, respectively. Corticosteroid or anti‐epileptic medication use was not reported. The Addenbrooke's Cognitive Examination III was used to evaluate cognition at baseline and following the 12‐week rehabilitation. Significant improvements were observed across all cognitive domains after 12 weeks in both the inpatient and outpatient groups, except for the language functioning subscale (*p* = 0.059) after the outpatient treatment program.

Hulshof et al.^37^ evaluated hyperbaric oxygen therapy in seven cognitively impaired adult patients that had received brain irradiation in a phase I/II randomised study at a single Dutch centre. Patients were randomised to receive either hyperbaric oxygen in 30 sessions delivered 5–6 times weekly (125 min/session), or delayed hyperbaric treatment. Tumour types included in the study were glioblastoma (28.6%), oligodendroglioma (14.3%), oligoastrocytoma (14.3%), ependymoma (14.3%), medulloblastoma (14.3%) and neuroblastoma (14.3%). Patients with a history of previous chemotherapy were excluded from the study. Rates of corticosteroid or anti‐epileptic medications were not reported. An extensive cognitive test battery evaluating processing speed, abstract reasoning, visual–spatial insight and visuoconstructive skills, object naming, verbal memory, vocabulary memory, memory for structured verbal material, numerical ability, nonverbal memory, executive functioning, selective attention, perceptual interference and response inhibition, cognitive flexibility, reaction time, and visual‐motor and speed coordination. Tests were performed at baseline and again at 3 and 6 months. Between baseline and the six‐month follow‐up assessment, three out of four of the patients in the immediate group, and all patients in the delayed group, demonstrated some improvement.

Locke et al.,^13^ in a uncontrolled/partially randomised single‐centre US trial, evaluated the effects of a two‐week cognitive rehabilitation program comprising of six 50‐min sessions compared with standard care. Of the 19 patients enrolled and randomised, outcome data were provided for 13. Of those enrolled, 10.5% had meningiomas and the rest (89.5%) had gliomas (specific subtypes were not specified). Radiotherapy and chemotherapy were noted in 94.7% and 63.2% of enrolled participants. Rates of corticosteroid or anti‐epileptic medications were not reported. Cognitive functioning was assessed using the R‐BANS but only reported at baseline and post‐intervention (and not at 3‐month follow‐up), as explained earlier. Control participants were more significantly impaired on a measure of immediate memory relative to the participants in the intervention group at baseline (*p* = 0.03), but no statistical comparisons were performed post‐intervention.

Maschio et al.,^17^ in an uncontrolled study, evaluated cognitive rehabilitation in patients with brain tumour‐related epilepsy and cognitive impairment recruited from a single centre in Italy. The intervention comprised of one weekly individual one‐hour session for 10 weeks, focusing on memory, attention, visuo‐spatial functions, language and reasoning. Twelve of the 16 participants enrolled completed the intervention. The tumour types included low‐grade glioma (31.3%), high‐grade glioma (25%), glioblastoma (12.5%), meningioma (12.5%) and metastasis (18.8%). Radiotherapy, chemotherapy, steroid medication and anti‐epileptic medication were reported in 68.8%, 31.3%, 12.5% and 100%, respectively. Tests evaluating global cognition, attention and executive functions, abstract reasoning, visuo‐spatial abilities, long‐term visuospatial memory, short‐term auditory‐verbal memory, long‐term auditory‐verbal memory and episodic memory, and language were evaluated at baseline, directly after the cognitive rehabilitation, and at six‐month follow‐up. At baseline, the greatest deficits observed were in both short‐ and long‐term verbal memory. At 6 months, all patients improved in at least one domain that was lower than normal at baseline.

Miotto et al.,^22^ in a nonrandomised function magnetic resonance imaging (fMRI) study, explored the brain correlates of 30 minutes of semantic strategy training and memory performance in 21 patients with distinct prefrontal cortex lesions recruited from a single centre in Brazil. Tumour types included astrocytoma (28.6%), olfactory groove meningioma (23.8%), other meningioma (19.0%), oligodendroglioma (23.8%) and glioblastoma (4.8%). Rates of radiotherapy, chemotherapy, corticosteroid, or anti‐epileptic drug use were not reported, but patients with a history of radiotherapy, chemotherapy, or drug treatment in the previous 6 months before and during the study were excluded. The study found improved word recall (F(1,22) = 109.75, *p* < 0.001) and semantic clustering (F(1,22) = 87.89, *p* < 0.001) after training. In a similar semantic strategy fMRI study by the same group, but with a healthy control group (*n* = 15) alongside 9 patients with left frontal low‐grade glioma resections,^43^ results varied according to whether words were semantically related or not.

Richard et al.,^18^ in a pilot randomised controlled trial, compared Goal Management Training (GMT), a behavioural intervention that combines mindfulness and strategy training, an active control condition (Brain Health Program, BHP) focusing on a supportive care intervention that offers education and activities promoting general brain health in the absence of cognitive strategy training, and a wait‐list control group (WAIT) in primary brain tumour patients recruited from a single Canadian centre. Of the 26 participants enrolled, 21 completed the study; noncompleters were reported to be younger at the point of enrolment and had worse language abilities, slower processing speed and higher apathy scores. Tumour types included meningioma (28%), low‐grade glioma (32%), high‐grade glioma (24%) and others (16%). Radiotherapy (partial or whole‐brain), chemotherapy (prior to or during the study), corticosteroid use during study and anti‐epileptic medication use during the study were reported in 85%, 44%, 0% and 44%, respectively. Cognitive function was assessed using measures covering executive function, memory and processing speed at baseline with a subset repeated immediately post‐training and 4 months later. A significant intervention effect was seen at 4 months on the Executive composite (time‐by‐group interaction: F(2,16) = 3.760, *p* = 0.046, ƞp2 = 0.320), that was reported to reflect a large improvement in scores in the GMT group (dw = 1.08, *p* = 0.002; dc = 1.09) and a minimal change of scores in both the BHP (dw = 0.09, *p* > 0.1) and WAIT (dw = −0.06, *p* > 0.1) groups. Results for memory and processing speed were less supportive of the superiority of GMT over the other conditions.

Sacks‐Zimmerman et al.^44^ investigated the efficacy of CogMed Working Memory Training (25 online training sessions, 30–45 min long) in three patients experiencing cognitive impairments post‐treatment for low‐grade glioma recruited from a single US centre. Tumour types included pilocytic astrocytoma (66.7%) and oligoastrocytoma (33.3%). Rates of radiotherapy, chemotherapy, corticosteroid use, or anti‐epileptic therapy were not reported. Cognitive tests encompassed measures of attention and memory, and were performed at baseline, within 2 weeks of training completion, and after 3 months. Results varied between the three patients and are nongeneralisable.

Schellart et al.,^20^ in an uncontrolled study, investigated the effect of hyperbaric oxygen therapy (five‐times weekly for 6–8 weeks) in 10 brain tumour patients recruited from a single Dutch centre that had received radiotherapy and surgery compared to 10 healthy matched controls. Tumour types included atypical meningioma (10%), anaplastic oligodendroglioma (10%), non‐Hodgkin lymphoma (20%), meningioma “en plaque” (10%), glioblastoma (10%), solitary brain metastasis (30%) and oligodendroglioma (10%). Chemotherapy was administered to 10% prior to the hyperbaric oxygen therapy. All patients received anti‐epileptic drugs, but the rates of corticosteroid use were not reported. Cognitive assessments, which comprised of a test of visual‐sensorimotor integrity and sustained attention, were performed 1 week before as well as 6 weeks and 4 months after completing the treatment. The results indicated that visual‐sensorimotor integrity and sustained attention were significantly improved in the brain tumour patients.

Taylor et al.,^14^ in an uncontrolled randomised single‐centre US study published as a conference abstract, investigated three cognitive rehabilitation strategies in terms of their feasibility and efficacy in 23 low‐grade glioma patients considered to have stable disease. Patients were randomised to receive in‐person rehabilitation, iPad‐based training, or daily instructional text messages. Previous radiotherapy was administered to 78% of participants, but the rates of chemotherapy, corticosteroid use and anti‐epileptic medication use were not reported. The specific cognitive tests used were not specified, but were administered at baseline and again 3 and 6 months following the intervention. The abstract only reported baseline data, which showed that the most commonly impaired cognitive domain was processing speed, with 43% of participants found to be ≥1.5 SD below normal.

Van der Linden et al.,^38^ in a randomised controlled trial, evaluated a tablet‐based cognitive rehabilitation program in patients with low‐grade glioma and meningioma recruited from three Dutch centres. Sixty‐two patients were randomised to cognitive rehabilitation or a wait‐list control group, and data on long‐term follow‐up were available for 45 of these. There were 55.1% participants with WHO grade I meningioma, 4.1% with WHO grade II meningioma, 38.8% with WHO grade II glioma and 2.0% with WHO grade III glioma. Following surgery, 28.6% and 20.4% received radiotherapy and chemotherapy, respectively. Use of psychotropic medication, defined in the study as anti‐epileptic drugs, corticosteroids, benzodiazepines, opioids, antipsychotics, stimulants and/or antidepressants, was reported in 57.1% of participants. Cognition was evaluated using a computerised battery of tests as well as tests of working memory and verbal fluency before surgery as well as 3, 6 and 12 months later. There was no significant difference in the group means over time for the performance‐based cognitive outcome measures. Furthermore, the reliable change indices between the intervention and control groups were not significantly different.

Voss et al.^21^ evaluated the effect of a calorie‐restricted ketogenic diet with intermittent fasting compared to standard diet in a randomised controlled trial of 50 patients with recurrent glioblastoma recruited from three German centres. A history of prior radiotherapy and concomitant chemotherapy was noted in 86% and 92% of participants, respectively, but rates of steroid or anti‐epileptic drug use were not reported. Twenty participants in the intervention group and 22 in the standard diet group completed the evaluation as set by the protocol. Cognitive functioning was assessed as a secondary outcome using the MMSE and the d6 Test of Attention at baseline and Day 6, Day 12 and 1 month following radiotherapy. Median MMSE scores were reported to be similar between baseline and follow‐up in both groups. d2 Test of Attention scores increased significantly until one‐month follow‐up in both arms and were not significantly different by treatment arm. Of note, the standard diet group had a lower‐than‐expected calorie intake (21 kcal per kg per day instead of 30 kcal per kg per day).

Yang et al.,^39^ in a randomised controlled trial, investigated virtual reality training (30 min daily, three times weekly) in combination with computer‐assisted cognitive rehabilitation (30 min daily, twice per week) compared to computer‐assisted cognitive rehabilitation (30 min daily, 5 days per week) alone for 4 weeks in 38 patients with brain tumours recruited from a single Korean centre. Tumour types included meningioma (26.3%), glioblastoma (13.2%), metastasis (15.8%), astrocytoma (5.3%) and others (39.5%; unspecified). Rates of radiotherapy, chemotherapy, steroid use and anti‐epileptic drug use were not reported. Cognition was assessed at baseline and again following 4 weeks of rehabilitation treatment using computerised neuropsychological tests of concentration, selective attention, verbal memory and spatial memory, as well as visual‐motor coordination and global cognitive function (Korean version of the MMSE). The study found that the virtual reality group had significantly improved scores in the domains of concentration, verbal memory, spatial memory and visual‐motor coordination.

Yu et al.^45^ retrospectively compared the effectiveness of intensive rehabilitation therapy (1 h each of physical and occupational therapy, 5 days per week during hospitalisation) for improving neurological deficits following brain tumour surgery (*n* = 35), including stroke patients (*n* = 108) as a reference group. Patients were treated at a single Korean centre, and the tumours were classified as WHO grades I (31.4%), II (28.6%), III (11.4%) and IV (28.6%), incorporating many tumour types without specific numbers of each. Radiotherapy and chemotherapy were administered to 54.3% and 31.4% of the brain tumour patients, respectively. Rates of corticosteroid and anti‐epileptic drug use were not reported. The occupational therapy sessions included cognitive ability enhancement. Cognition was assessed using a global measure of cognition along with intelligence at baseline and more than 1 month after admission. Significant improvements in global cognition and intelligence scores were identified in both the brain tumour and stroke groups (both ps < 0.001) and within benign and malignant brain tumour subgroups (both ps < 0.01). No significant between‐group or between‐subgroup differences were observed in effectiveness or efficiency.

Zucchella et al.^15^ performed a randomised trial comparing cognitive rehabilitation (four one‐hour sessions per week for 4 weeks) to standard rehabilitative care without cognitive training in 53 early post‐surgical brain tumour patients recruited from a single centre in Italy. Tumour types included high‐grade glioma (47.2%), low‐grade glioma (13.2%), meningioma (30.2%) and others (9.4%; unspecified). None of the patients had received radiotherapy or chemotherapy prior to or during the study, but 22.6% and 37.7% were taking corticosteroids and anti‐epileptic medications, respectively. A range of validated cognitive tests were performed encompassing global cognitive function, verbal and spatial immediate memory span, verbal memory (immediate and delayed recall), nonverbal reasoning, frontal functionality, simple speed processing and complex attention, visual selective attention, visuo‐constructional abilities and verbal fluency, which were performed at baseline (within 3 days of admission) and after 4 weeks. The study reported significant improvements in the rehabilitation group, particularly in verbal memory and visual attention, and nonsignificant improvements in the control group.

## 4 DISCUSSION

This systematic review of interventions for cognition in adult patients with brain tumours identified 35 randomised and nonrandomised studies describing a range of pharmacological and nonpharmacological interventions. Although side effects or adverse events were not reported in all studies identified in this review, when reported the rates and severity of such complications were both low.

Of the 14 pharmacological studies that were included in this systematic review, supportive evidence was found for memantine,^7^ ginkgo biloba^35^ and shenqi fuzheng^34^ for the prevention of cognitive deficits in patients with brain tumours. There was also supportive evidence for a role for donepezil,^8^, ^9^ methylphenidate^5^, ^36^ and modafinil^4^, ^5^ in the amelioration of pre‐existing cognitive impairment. Some of these studies were at high risk of bias. For example: The study of modafinil by Kaleita et al.^4^ was a conference abstract with a corresponding lack of detail about the study methodology and only eight‐week follow‐up data; the study by Gehring et al.^5^ comparing two doses of methylphenidate to modafinil was characterised by an open‐label design, a high proportion of drop‐out, and employed an exploratory statistical analysis approach combining the two doses of methylphenidate due to low accrual; and it was not possible for participant blinding to occur in the study by Chen et al.^34^ as the comparator groups were shenqi fuzheng injection with radiotherapy versus radiotherapy alone. Furthermore, the primary outcomes for the two randomised studies of memantine^7^ and donepezil^8^ were nonsignificant, with only secondary outcomes that were significant; caution is required when interpreting secondary outcome results alongside nonsignificant results in the primary outcome measure(s).

A number of the 21 nonpharmacological intervention studies identified positive results, even if sometimes improvements were noted in some cognitive domains but not others. A range of rehabilitation interventions, including specialist neurorehabilitation and general rehabilitation, were shown to be positively associated with the prevention^11^, ^12^, ^16^, ^17^, ^42^, ^45^ or amelioration^15^ of cognitive impairment. Positive findings were also identified for working memory training,^40^ Goal Management Training,^18^ aerobic exercise,^19^ virtual reality training combined with computer‐assisted cognitive rehabilitation,^39^ and hyperbaric oxygen therapy^20^, ^37^ in the amelioration of cognitive impairment, and semantic strategy training^22^, ^43^ in the prevention of cognitive impairment. A number of the studies were at high risk of bias and non‐randomised in design. For example, Durà Mata et al.^11^ published a conference abstract that did not specify the cognitive tests or statistical analyses performed and blinding was not possible due to the nature of the intervention (cognitive telerehabilitation). Gehring et al.^19^ compared aerobic exercise to an active control condition, but the study was limited by a lack of blinding of both the participants and cognitive outcome assessors. The study by Voss et al.^21^ comparing ketogenic diet and intermittent fasting to standard diet used inadequate cognitive assessment measures was missing cognitive outcome data for a large proportion of participants, had a short follow‐up assessment period (1 month), and the standard diet group had an average calorie intake level similar to the intervention arm. Yang et al.,^39^ comparing virtual reality training and computer‐assisted cognitive rehabilitation compared to computer‐assisted cognitive rehabilitation alone in a randomised study, did not describe the randomisation process, participant blinding was not possible due to the nature of the intervention, data were not presented on whether outcome data were presented for all participants randomised, and it was not specified whether blinding of outcome assessors took place.

Although a number of positive results were identified by this review, most studies were deemed to be at moderate‐to‐high risk of bias, and there was wide heterogeneity in type and delivery of interventions, patient populations (including tumour types and treatments received), type and timing of outcome measures administered, and outcome reporting. The randomised studies were most commonly at risk of bias due to open‐label study designs and lack of blinding, missing outcome data, and a lack of use of a battery of cognitive tests to evaluate multiple cognitive domains. The nonrandomised studies were deemed to be at risk of bias due to multiple measurements not being obtained prior to the intervention, small sample sizes, a lack of enrolment of all eligible participants, and a high loss of follow‐up after baseline assessments. Most studies did not report effect sizes, limiting the ability to interpret the strength of any relationships identified.

Although our review has identified several positive findings, there remains uncertainty around the durability of any benefits on cognition once the intervention ends, particularly in relation to rehabilitation interventions. This may explain why few clinical practice guidelines have incorporated recommendations for the use of specific rehabilitation interventions,^46^ for example. It is important to highlight that some of the weaknesses of the included studies reflect the challenges of conducting studies in patients with brain tumours; low accrual, sample heterogeneity, and high drop‐out levels have all been observed in some of the identified studies. There are myriad influences on cognitive outcomes in patients with brain tumours, including patient‐, treatment‐ and tumour‐specific factors, and there are factors that are likely important but relatively understudied, such as cognitive reserve (individual differences in task processing that permit some to cope with a brain condition better than others), brain reserve (individual differences in the brain itself that allow some to cope better than others with a given condition), and socioeconomic status.^4^ With so many potential influencing variables, it is difficult to sufficiently account for the resulting heterogeneity in small, underpowered studies that struggle to recruit sufficient numbers of participants due to the aforementioned challenges associated with studies of this population group. Larger, multicentre studies could help standardise methods and outcome measures, potentially leading to more robust and generalisable findings.

Some of the identified studies found greater improvements in cognition post‐intervention in those who were more cognitively impaired at baseline,^5^, ^8^ and thus, future studies may wish to target specific “at‐risk” populations in order to realise the benefits of any interventions studied. Whether reported improvements in cognitive test scores translate into clinically relevant, sustained long‐term benefits on a patient's everyday life are difficult to ascertain; future studies should consider more detailed correlations between subjective and objective cognitive performance over a longer follow‐up period to elucidate on this further.

There are several limitations to discuss in the context of this systematic review. There was wide heterogeneity in the characteristics of participants included in the studies and in the employed methodologies of the identified studies, with myriad cognitive tests used, outcome assessments performed at varying time points in relation to treatments, and varied follow‐up lengths. Some of the included studies relied on one or two measures of cognitive function, including global measures such as the MMSE, which is not able to comprehensively assess cognitive function, and evidence indicates that it is only possible to obtain reliable results with the use of a comprehensive neuropsychological battery, as such screening tools are not able to detect all instances of cognitive dysfunction.^47^ A reasonable proportion of the included studies included multiple pathologies with no subgroup analysis by tumour type or grade; there is evidence from noninterventional studies that cognitive outcomes vary by tumour type even when considering tumours located in the same region anatomically.^48^ Reporting of variables that likely influence cognitive outcomes, including anti‐epileptic and steroid use, was inconsistent across studies. Many of the studies had withdrawal/drop‐out rates >5%, and it was often difficult to establish whether the participants who withdrew/dropped out of the studies differed in baseline characteristics and had worse prognoses. High drop‐out rates are not uncommon in studies of patients with tumours, due to disease progression and other factors. Although the search strategy was broad to attempt to identify as many potentially relevant papers as possible, we may still have missed eligible studies. Finally, it was not possible to fully appraise all included studies as, although authors of all studies included in this study that required clarification were contacted, only a proportion responded.

## 5 CONCLUSIONS

This systematic review of interventions for cognition in adult patients with brain tumours identified 35 randomised and nonrandomised studies meeting the inclusion/exclusion criteria of this review. A range of pharmacological and nonpharmacological interventions for the amelioration or prevention of cognitive impairment were described. Of these, a number of pharmacological interventions were found to be associated with positive effects on cognition, including pharmacological agents such as memantine, donepezil, methylphenidate, modafinil, ginkgo biloba and shenqi fuzheng. Similarly, the nonpharmacological interventions of general and cognitive rehabilitation, working memory training, Goal Management Training, aerobic exercise, virtual reality training combined with computer‐assisted cognitive rehabilitation, hyperbaric oxygen therapy and semantic strategy training were also associated with positive effects on cognition. Despite this, caution is required as most identified studies had a number of limitations and were deemed to be at moderate‐to‐high risk of bias. Some of the benefits associated with interventions were identified in nonrandomised studies, and such interventions should be evaluated in a randomised trial setting where possible. In addition, it remains unclear whether and to what extent the identified interventions lead to durable cognitive benefits after cessation of the intervention. Further studies should focus on improved study reporting (including the use of medications such as corticosteroids and anti‐epileptics) and methods (including blinding of outcome assessors) to reduce bias, methods to minimise participant drop‐out and withdrawal where possible, and consider standardisation of methods and interventions across studies. Greater collaboration between centres could result in larger studies with standardised methods and outcome measures, and should be a focus of future research efforts in the field.

## Funding information

Matthew A. Kirkman is supported by a studentship from the Economic and Social Research Council awarded via University College London (studentship reference: 1924548; grant reference: ES/P000592/1). For the purpose of open access, the author has applied a Creative Commons Attribution (CC BY) licence to any Author Accepted Manuscript version arising.

## AUTHOR CONTRIBUTIONS

**Matthew Anthony Kirkman:** Conceptualization (equal); data curation (equal); formal analysis (equal); funding acquisition (equal); investigation (equal); writing – original draft (equal); writing – review and editing (equal). **Justyna O Ekert:** Data curation (equal); formal analysis (equal); writing – review and editing (equal). **Ben HM Hunn:** Data curation (equal); writing – review and editing (equal). **Michael SC Thomas:** Conceptualization (equal); supervision (equal); writing – review and editing (equal). **Andrew K Tolmie:** Conceptualization (equal); supervision (equal); writing – review and editing (equal).

## Supporting information


Appendix S1

Appendix S2

Appendix S3

## Data Availability

The data that support the findings of this study are available from the corresponding author upon reasonable request.
