# Supplementary material for: A systematic review of cognitive interventions for adult patients with brain tumours
Source: Cancer Med. 2023 Mar 7;12(10):11191–210. doi: 10.1002/cam4.5760 (PMC10242361; doi:10.1002/cam4.5760)
Supplement: Supplementary file 2 — Appendix S2 [file CAM4-12-11191-s002.docx]

A systematic review of cognitive interventions for adult patients with brain tumours

Authors: Matthew A. Kirkman,^1,2^ Justyna O. Ekert,^3^ Benjamin H.M. Hunn,^4,5,6^ Michael S.C. Thomas,^7^ Andrew K. Tolmie^1^

1. Department of Psychology and Human Development, UCL Institute of Education, University College London, London, UK

2. Department of Neurosurgery, Queen’s Medical Centre, Nottingham University Hospitals NHS Trust, Nottingham, UK

3. Wellcome Centre for Human Neuroimaging, UCL Queen Square Institute of Neurology, London, UK

4. Department of Neurosurgery, Royal Melbourne Hospital, Melbourne, Australia

5. Department of Neurosurgery, Royal Hobart Hospital, Hobart, Australia

6. School of Medicine, University of Tasmania, Hobart, Australia

7. Department of Psychological Sciences, Birkbeck, University of London, London, UK

Running title: Cognitive interventions for brain tumours

*Corresponding author:

Matthew A. Kirkman

Department of Psychology and Human Development

UCL Institute of Education

University College London

25 Woburn Square

London WC1H 0AA

UK

Email: matthew.kirkman.17@ucl.ac.uk

Phone: + 44 7886608978

| **Search themes** | **Search term(s)** |
| --- | --- |
| Brain tumour | Brain neoplasms.mp. or exp Brain Neoplasms/ |
|  | exp Astrocytoma/ or Neoplasms, Neuroepithelial.mp. or exp Medulloblastoma/ or  exp Cerebellar Neoplasms/ or exp Neoplasms, Neuroepithelial/ or exp Neuroectodermal Tumors, Primitive, Peripheral/ or exp Brain Neoplasms/ |
|  | Neoplasm Metastasis.mp. or exp Neoplasm Metastasis/ |
|  | (Brain tum* or cerebral tum* or glio* or brain metastas* or cerebral metastas* or supratentorial tum* or supratentorial metastas* or infratentorial tum* or infratentorial metastas* or brain cancer or cerebral cancer or brain malignan* or cerebral malignan* or primary brain tum* or primary brain cancer or brain neoplas* or cerebral neoplas* or suptratentorial neoplas* or infratentorial neoplas*).mp. |
|  | Meningioma/ or intracranial tumor.mp. or Pituitary Neoplasms/ or Meningeal  Neoplasms/ |
|  | Skull Base/ or Skull Neoplasms/ or Skull Base Neoplasms/ or skull base tumor.mp. |
|  |  |
| Cognition | Mental processes.mp. or exp Mental Processes/ |
|  | Neurobehavioral manifestation.mp. or exp Neurobehavioral Manifestations/ |
|  | Psychological tests.mp. or exp Psychological Tests/ |
|  | Neuropsychology.mp. or exp Neuropsychology/ |
|  | Neuropsychiatry.mp. or exp Neuropsychiatry/ |
|  | Neurocognitive disorders.mp. or exp Neurocognitive Disorders/ |
|  | Psychomotor performance.mp. or exp Psychomotor Performance/ |
|  | Cognitive neuroscience.mp. or exp Cognitive Neuroscience/ |
|  | (Cogniti* or psycholog* or neuropsycholog* or memory or executive functio* or spatia* or learnin* or percept* or attentio* or intell* or neuropsychiat* or psychologica* or behavio* or neurocogniti*).mp. |
|  |  |
| Outcome/ recovery/ plasticity | Outcome assessment health care.mp. or exp “Outcome Assessment (Health Care)”/ |
|  | (Recover* or outcom* or plastic*).mp. |
|  |  |

**Online Resource 2**: Search strategy used in this systematic review
