# Supplementary material for: A systematic review of cognitive interventions for adult patients with brain tumours
Source: Cancer Med. 2023 Mar 7;12(10):11191–210. doi: 10.1002/cam4.5760 (PMC10242361; doi:10.1002/cam4.5760)
Supplement: Supplementary file 3 — Appendix S3 [file CAM4-12-11191-s001.docx]

A systematic review of cognitive interventions for adult patients with brain tumours

Authors: Matthew A. Kirkman,^1,2^ Justyna O. Ekert,^3^ Benjamin H.M. Hunn,^4,5,6^ Michael S.C. Thomas,^7^ Andrew K. Tolmie^1^

1. Department of Psychology and Human Development, UCL Institute of Education, University College London, London, UK

2. Department of Neurosurgery, Queen’s Medical Centre, Nottingham University Hospitals NHS Trust, Nottingham, UK

3. Wellcome Centre for Human Neuroimaging, UCL Queen Square Institute of Neurology, London, UK

4. Department of Neurosurgery, Royal Melbourne Hospital, Melbourne, Australia

5. Department of Neurosurgery, Royal Hobart Hospital, Hobart, Australia

6. School of Medicine, University of Tasmania, Hobart, Australia

7. Department of Psychological Sciences, Birkbeck, University of London, London, UK

Running title: Cognitive interventions for brain tumours

*Corresponding author:

Matthew A. Kirkman

Department of Psychology and Human Development

UCL Institute of Education

University College London

25 Woburn Square

London WC1H 0AA

UK

Email: matthew.kirkman.17@ucl.ac.uk

Phone: + 44 7886608978

**Online Resource 3:** Risk of Bias assessments

### Methods

The risk of bias assessments were performed independently by two authors (MAK and JOE), and disagreements were resolved through consensus. Where there was an unclear risk of bias, authors were contacted for clarification via email.

The Cochrane Risk of Bias 2 assessment tool [1] was used to assess the risk of bias among the randomised studies. This evaluates the risk of bias in five domains (randomisation process, deviations from intended interventions, missing outcome data, measurement of the outcome, and selection of the reported result), resulting in an overall risk of bias assessment in addition to individual domain assessments. The risk of bias judgment is graded as ‘high’, ‘low’, ‘some concerns’ or ‘no information’. The guidance notes and algorithm provided at https://www.riskofbias.info/welcome/rob-2-0-tool were used for ratings, with deviations from the algorithm-determined ratings agreed between the two authors performing the risk of bias assessments (MAK and JOE).

For non-randomised studies, the National Heart, Lung, and Blood Institute (NHLBI) Quality Assessment Tool for Before-After (Pre-Post) Studies With No Control Group (available at: https://www.nhlbi.nih.gov/health-topics/study-quality-assessment-tools) was used to evaluate risk of bias. This comprises of 12 questions evaluating studies, with the 12 questions covering the domains of: study question; eligibility criteria and study population; study participants representative of clinical populations of interest; whether all eligible participants were enrolled; sample size; whether the intervention was clearly described; whether the outcome measures were clearly described, valid and reliable; blinding of outcome assessors; follow-up rate; statistical analysis; use of multiple measurements of outcomes; and group-level interventions and individual-level outcome efforts. Responses to these domains are graded as ‘Yes’, ‘No’, or ‘Other’ (cannot determine, not applicable, or not reported), and an overall quality assessment rating of ‘Good’, ‘Fair’, or ‘Poor’. Due to the lack of a standardised criteria or algorithm for grading, we devised the following thresholds: studies with one or less of the 12 domains rated as ‘No’, ‘Cannot Determine’ or ‘Not Reported’ were rated as ‘Good’ overall; studies with between two and five of the 12 domains rated as ‘No’, ‘Cannot Determine’ or ‘Not Reported’ were rated as ‘Fair’ overall; and studies with six or more of the 12 domains rated as ‘No’, ‘Cannot Determine’ or ‘Not Reported’ were rated as ‘Poor’ overall.

Risk-of-bias plots were created using the *robvis* tool [2].

### Results

As the risk of bias tools used were contingent on the study design, the results are separated according to whether the study was randomised (assessed using the Cochrane Risk of Bias 2 tool) or non-randomised (assessed using the NHLBI Quality Assessment Tool for Before-After (Pre-Post) Studies With No Control Group). Results of the risk of bias assessments are described below and in Figures 2–5, which show that many of the included studies were at high risk of bias.

#### Randomised studies

Of the 22 randomised studies evaluated using the Cochrane Risk of Bias 2 tool, only four were found to have a low risk of bias overall (Figure 2) [3-6]. Thirteen of the 20 studies were found to have a high risk of bias overall [7-19], and five with some concerns overall [20-24]. The domain with the most ‘high-risk’ ratings across all studies was ‘bias in measurement of the outcome’ (41% of the 22 randomised studies), followed by ‘bias due to missing outcome data’ (36%), ‘bias due to deviations from intended interventions’ (32%), ‘bias arising from the randomisation process’ (23%), and ‘bias in selection of the reported result’ (9%; Figure 3).

Regarding domain 1, bias arising from the randomisation process, five studies were considered ‘high risk’ [8-10, 13, 17]: two due to an open-label design [9, 10] and one due to a lack of blinding of researchers who assigned participants to the intervention [17]. In one study, some participants were not randomised due to low study accrual and allocated directly to an intervention group [13]. No detailed information was provided about the randomisation process in one study [8].

There were seven high-risk studies for domain 2 (bias due to deviations from intended intervention) [7-12, 16]. This was due to the study having an open-label design or it being impossible to blind participants due to the nature of the intervention [7-11, 16] and lack of information provided about deviations from the intended intervention that may have arisen due to the trial context [7, 8, 12, 16].

High risk of bias due to missing outcome data, characterised in domain 3, was identified in eight studies [7-9, 12, 14, 16, 18, 19]. Data were reported as missing for ≤5% participants in 3 (14%) of the 22 randomised studies, >5% participants in 14 (64%) studies, and no information was provided on the proportion of missing data for five (23%) studies. Eleven of the 14 studies with >5% missing outcome data were not classified as ‘high risk’ as there was evidence that the results were not biased by missing data [3-6] or the risk/likelihood that the missingness of the data depended on its true value was low [11, 13, 15, 17, 22-24]. Five studies provided no information relating to the domain and were thus classified as high risk of bias [7, 8, 12, 16, 19].

Nine studies were evaluated as being high risk in relation to measurement of the outcome (domain 4) [8, 13, 15, 18, 23]. In five of these studies, only one or two cognitive tests were used to evaluate cognitive function, insufficient to fully evaluate cognitive function [8, 13, 15, 18, 23]; for example, the Butler et al. [23] study relied on the MMSE alone as an outcome measure, which is unlikely to provide a valid and comprehensive assessment of cognition on its own [25]. Furthermore, in one of these studies [13] no follow-up cognitive data were presented as most participants did not return in-person for follow up, precluding them from completing the cognitive test used in that study (R-BANS). Two studies were considered high risk due to the outcome assessors being aware of the intervention received and it was felt likely that knowledge of the intervention influenced outcome assessment [10, 11], and in three studies insufficient information was provided about the blinding status of outcome assessors [7, 8, 12].

Only two studies were classified as high risk for domain 5 of the Risk of Bias 2 tool, relating to bias in selection of the reported result [13, 15]. For one of the two studies [15], there was a discrepancy between the ClinicalTrials.gov entry and manuscript regarding the outcome measures reported, and the manuscript stated the use of post hoc analyses. The other study, by Locke et al. [13], was considered high risk due to the aforementioned issues relating to the lack of follow-up cognitive data being reported. There were some concerns in this domain for 12 (55%) of the 22 randomised studies.

#### Non-randomised studies

Of the 13 non-randomised studies included in this systematic review, three were assessed as having an overall rating of ‘poor’ using the NHLBI Quality Assessment Tool for Before-After (Pre-Post) Studies With No Control Group [26-28], and the remainder were assessed as having an overall rating of ‘fair’ (Figure 4) [29-38]; thus, none of the included studies were evaluated as ‘good’ overall.

Across the 12 questions addressed using this tool, the highest proportion of ‘No’ responses (indicating a higher risk of bias) were found in response to question 11 (“Were outcome measures of interest taken multiple times before the intervention and multiple times after the intervention (i.e., did they use an interrupted time-series design)?”) in 13 (100%) studies, as none of the studies performed outcome assessments more than once before the intervention (Figure 5). The question with the second highest ‘No’ response was question 5 (“Was the sample size sufficiently large to provide confidence in the findings?”) in nine (69.2%) studies, followed by question 4 (“Were all eligible participants that met the prespecified entry criteria enrolled?”) in 6 (46.2%) studies, and question 9 (“Was the loss to follow-up after baseline 20% or less? Were those lost to follow-up accounted for in the analysis?”) in 5 (38.5%) studies. Only one question, question 6 (“Was the test/service/intervention clearly described and delivered consistently across the study population?”) had a response of ‘Yes’ across all 13 studies. All but one study was evaluated as ‘Yes’ in response to question 7 (“Were the outcome measures prespecified, clearly defined, valid, reliable, and assessed consistently across all study participants?”) [38]. Question 12 (“If the intervention was conducted at a group level (e.g., a whole hospital, a community, etc.) did the statistical analysis take into account the use of individual-level data to determine effects at the group level?”) was determined as being not applicable to any of the studies. None of the remaining questions were deemed ‘not applicable’ in any of the 13 studies.

### References

1. Sterne JAC, Savović J, Page MJ, et al (2019) RoB 2: a revised tool for assessing risk of bias in randomised trials. BMJ 366:l4898. doi: 10.1136/bmj.l4898

2. McGuinness LA, Higgins JPT (2021) Risk-of-bias VISualization (robvis): An R package and Shiny web app for visualizing risk-of-bias assessments. Res Synth Methods 12:55–61. doi: 10.1002/jrsm.1411

3. Boele FW, Douw L, de Groot M, et al (2013) The effect of modafinil on fatigue, cognitive functioning, and mood in primary brain tumor patients: a multicenter randomized controlled trial. Neuro-oncology 15:1420–1428. doi: 10.1093/neuonc/not102

4. Porter AB, Liu H, Kohli S, et al (2022) Efficacy of Treatment With Armodafinil for Cancer-Related Fatigue in Patients With High-grade Glioma: A Phase 3 Randomized Clinical Trial. JAMA Oncol 8:259–267. doi: 10.1001/jamaoncol.2021.5948

5. Rapp SR, Case LD, Peiffer A, et al (2015) Donepezil for Irradiated Brain Tumor Survivors: A Phase III Randomized Placebo-Controlled Clinical Trial. Journal of Clinical Oncology 33:1653–1659. doi: 10.1200/JCO.2014.58.4508

6. Brown PD, Pugh S, Laack NN, et al (2013) Memantine for the prevention of cognitive dysfunction in patients receiving whole-brain radiotherapy: a randomized, double-blind, placebo-controlled trial. Neuro-oncology 15:1429–1437. doi: 10.1093/neuonc/not114

7. Durà Mata MJ, Molleda Marzo M, Teixidor P, et al (2018) Randomized controlled trial on the impact of cognitive telerehabilitation on cognition and quality of life in glioma patients. Ann Phys Rehabil Med 61:e271.

8. Chen L-J, Zhang R-G, Yu D-D, et al (2019) Shenqi Fuzheng Injection Ameliorates Radiation-induced Brain Injury. Curr Med Sci 39:965–971. doi: 10.1007/s11596-019-2129-9

9. Gehring K, Patwardhan SY, Collins R, et al (2012) A randomized trial on the efficacy of methylphenidate and modafinil for improving cognitive functioning and symptoms in patients with a primary brain tumor. J Neurooncol 107:165–174. doi: 10.1007/s11060-011-0723-1

10. Gehring K, Stuiver MM, Visser E, et al (2020) A pilot randomized controlled trial of exercise to improve cognitive performance in patients with stable glioma: a proof of concept. Neuro-oncology 22:103–115. doi: 10.1093/neuonc/noz178

11. Hulshof MCCM, Stark NM, van der Kleij A, et al (2002) Hyperbaric oxygen therapy for cognitive disorders after irradiation of the brain. Strahlenther Onkol 178:192–198. doi: 10.1007/s00066-002-0916-9

12. Kaleita TA, Wellisch DK, Graham CA, et al (2006) Pilot study of modafinil for treatment of neurobehavioral dysfunction and fatigue in adult patients with brain tumors. Journal of Clinical Oncology 24:1503.

13. Locke DEC, Cerhan JH, Wu W, et al (2008) Cognitive rehabilitation and problem-solving to improve quality of life of patients with primary brain tumors: a pilot study. J Support Oncol 6:383–391.

14. Page BR, Shaw EG, Lu L, et al (2015) Phase II double-blind placebo-controlled randomized study of armodafinil for brain radiation-induced fatigue. Neuro-oncology 17:1393–1401. doi: 10.1093/neuonc/nov084

15. Peng Y, Zhang W, Zhou X, et al (2016) Lidocaine Did Not Reduce Neuropsychological-Cognitive Decline in Patients 6 Months After Supratentorial Tumor Surgery: A Randomized, Controlled Trial. J Neurosurg Anesthesiol 28:6–13. doi: 10.1097/ANA.0000000000000171

16. Taylor J, Weyer-Jamora C, Brie M, et al (2020) NCOG-21. Interim results of three cognitive rehabilitation strategies in patients with lower grade gliomas. Neuro-oncology 22:ii133–ii134.

17. van der Linden SD, Rutten G-JM, Dirven L, et al (2021) eHealth cognitive rehabilitation for brain tumor patients: results of a randomized controlled trial. J Neurooncol 154:315–326. doi: 10.1007/s11060-021-03828-1

18. Voss M, Wenger KJ, Mettenheim von N, et al (2022) Short-term fasting in glioma patients: analysis of diet diaries and metabolic parameters of the ERGO2 trial. Eur J Nutr 61:477–487. doi: 10.1007/s00394-021-02666-1

19. Yang S, Chun MH, Son YR (2014) Effect of virtual reality on cognitive dysfunction in patients with brain tumor. Ann Rehabil Med 38:726–733. doi: 10.5535/arm.2014.38.6.726

20. Gehring K, Sitskoorn MM, Gundy CM, et al (2009) Cognitive rehabilitation in patients with gliomas: a randomized, controlled trial. Journal of Clinical Oncology 27:3712–3722. doi: 10.1200/JCO.2008.20.5765

21. Richard NM, Bernstein LJ, Mason WP, et al (2019) Cognitive rehabilitation for executive dysfunction in brain tumor patients: a pilot randomized controlled trial. J Neurooncol 142:565–575. doi: 10.1007/s11060-019-03130-1

22. Zucchella C, Capone A, Codella V, et al (2013) Cognitive rehabilitation for early post-surgery inpatients affected by primary brain tumor: a randomized, controlled trial. J Neurooncol 114:93–100. doi: 10.1007/s11060-013-1153-z

23. Butler JM, Case LD, Atkins J, et al (2007) A phase III, double-blind, placebo-controlled prospective randomized clinical trial of d-threo-methylphenidate HCl in brain tumor patients receiving radiation therapy. Radiation Oncology Biology 69:1496–1501. doi: 10.1016/j.ijrobp.2007.05.076

24. Laigle-Donadey F, Ducray F, Boone M, et al (2019) A phase III double-blind placebo-controlled randomized study of dexamphetamine sulfate for fatigue in primary brain tumors patients: An ANOCEF trial (DXA). Neuro-Oncology Advances 1:vdz043. doi: 10.1093/noajnl/vdz043

25. Racine CA, Li J, Molinaro AM, et al (2015) Neurocognitive Function in Newly Diagnosed Low-grade Glioma Patients Undergoing Surgical Resection With Awake Mapping Techniques. Neurosurgery 77:371–9– discussion 379. doi: 10.1227/NEU.0000000000000779

26. Hojan K, Gerreth K (2020) Can Multidisciplinary Inpatient and Outpatient Rehabilitation Provide Sufficient Prevention of Disability in Patients with a Brain Tumor?-A Case-Series Report of Two Programs and A Prospective, Observational Clinical Trial. Int J Environ Res Public Health 17:6488. doi: 10.3390/ijerph17186488

27. Sacks-Zimmerman A, Duggal D, Liberta T (2015) Cognitive Remediation Therapy for Brain Tumor Survivors with Cognitive Deficits. Cureus 7:e350. doi: 10.7759/cureus.350

28. Schellart NAM, Reits D, van der Kleij AJ, Stalpers LJA (2011) Hyperbaric oxygen treatment improved neurophysiologic performance in brain tumor patients after neurosurgery and radiotherapy: a preliminary report. Cancer 117:3434–3444. doi: 10.1002/cncr.25874

29. Attia A, Rapp SR, Case LD, et al (2012) Phase II study of Ginkgo biloba in irradiated brain tumor patients: effect on cognitive function, quality of life, and mood. J Neurooncol 109:357–363. doi: 10.1007/s11060-012-0901-9

30. Shaw EG, Rosdhal R, D'Agostino RB, et al (2006) Phase II study of donepezil in irradiated brain tumor patients: effect on cognitive function, mood, and quality of life. Journal of Clinical Oncology 24:1415–1420. doi: 10.1200/JCO.2005.03.3001

31. Meyers CA, Weitzner MA, Valentine AD, Levin VA (1998) Methylphenidate therapy improves cognition, mood, and function of brain tumor patients. J Clin Oncol 16:2522–2527. doi: 10.1200/JCO.1998.16.7.2522

32. Braun SE, Aslanzadeh FJ, Lanoye A, et al (2021) Working memory training for adult glioma patients: a proof-of-concept study. J Neurooncol 155:25–34. doi: 10.1007/s11060-021-03839-y

33. Han EY, Chun MH, Kim BR, Kim HJ (2015) Functional Improvement After 4-Week Rehabilitation Therapy and Effects of Attention Deficit in Brain Tumor Patients: Comparison With Subacute Stroke Patients. Ann Rehabil Med 39:560–569. doi: 10.5535/arm.2015.39.4.560

34. Hassler MR, Elandt K, Preusser M, et al (2010) Neurocognitive training in patients with high-grade glioma: a pilot study. J Neurooncol 97:109–115. doi: 10.1007/s11060-009-0006-2

35. Maschio M, Dinapoli L, Fabi A, et al (2015) Cognitive rehabilitation training in patients with brain tumor-related epilepsy and cognitive deficits: a pilot study. J Neurooncol 125:419–426. doi: 10.1007/s11060-015-1933-8

36. Miotto EC, Balardin JB, Vieira G, et al (2014) Right inferior frontal gyrus activation is associated with memory improvement in patients with left frontal low-grade glioma resection. PLoS ONE 9:e105987. doi: 10.1371/journal.pone.0105987

37. Miotto EC, Savage CR, Evans JJ, et al (2013) Semantic strategy training increases memory performance and brain activity in patients with prefrontal cortex lesions. Clinical Neurology and Neurosurgery 115:309–316. doi: 10.1016/j.clineuro.2012.05.024

38. Yu J, Jung Y, Park J, et al (2019) Intensive Rehabilitation Therapy Following Brain Tumor Surgery: A Pilot Study of Effectiveness and Long-Term Satisfaction. Ann Rehabil Med 43:129–141. doi: 10.5535/arm.2019.43.2.129
